# Identity crisis in alchemical space drives the entropic colloidal glass transition

**DOI:** 10.1038/s41467-018-07977-2

**Published:** 2019-01-08

**Authors:** Erin G. Teich, Greg van Anders, Sharon C. Glotzer

**Affiliations:** 1 Applied Physics Program, University of Michigan, Ann Arbor, MI 48109 USA; 2 Department of Physics, University of Michigan, Ann Arbor, MI 48109 USA; 3 Department of Chemical Engineering, University of Michigan, Ann Arbor, MI 48109 USA; 4 Department of Materials Science and Engineering, University of Michigan, Ann Arbor, MI 48109 USA; 5 Biointerfaces Institute, University of Michigan, Ann Arbor, MI 48109 USA

## Abstract

A universally accepted explanation for why liquids sometimes vitrify rather than crystallize remains hotly pursued, despite the ubiquity of glass in our everyday lives, the utilization of the glass transition in innumerable modern technologies, and nearly a century of theoretical and experimental investigation. Among the most compelling hypothesized mechanisms underlying glass formation is the development in the fluid phase of local structures that somehow prevent crystallization. Here, we explore that mechanism in the case of hard particle glasses by examining the glass transition in an extended alchemical (here, shape) space; that is, a space where particle shape is treated as a thermodynamic variable. We investigate simple systems of hard polyhedra, with no interactions aside from volume exclusion, and show via Monte Carlo simulation that glass formation in these systems arises from a multiplicity of competing local motifs, each of which is prevalent in—and predictable from—nearby ordered structures in alchemical space.

## Abstract (web summary)

Fluids may avoid crystallization via an underlying mechanism that remains hotly debated. Teich et al. show that hard polyhedral particles form glass because of the competition of local structural motifs, each of which is prevalent in crystals self-assembled from particles of closely related shapes.

## Introduction

Fluids, upon rapid cooling or compression, may bypass crystallization and instead remain disordered, displaying relaxation times that grow by orders of magnitude over small density or temperature windows. This fall out of equilibrium is widely known throughout the scientific community as the glass transition, and leveraged in many modern technologies including rewritable data storage devices^1^ and fiber optic networks^2^. Despite this widespread use and a general agreement in the research community on the phenomenological behavior of fluids as they are super-cooled or super-compressed, the underlying physical mechanism of the glass transition remains in contention. New, competing ideas—including revived versions of old ideas—continue to emerge^3,4^. In particular, the identification of local structure in various glass-forming systems and the establishment of its relationship to crystallization failure is an ongoing endeavor^5,6^. What these local structures are, why they form, and how exactly they prevent long-range ordering are central associated questions.

Prior works have considered individual systems, and argued that certain local structures arise in specific systems because they are preferred on a local length scale, but are prevented from growing and converting the liquid to a crystal because the structures are incommensurate with the embedding space^5,7–9^, arrange themselves non-periodically^10–14^, or are numerous in type and random in arrangement^15,16^. These works, however, do not consider the system under investigation in the context of other closely-related systems. Another pool of studies^17–24^ views vitrification more explicitly as the structural frustration of emerging crystalline order. In that context, local bond-orientational ordering and multiple medium-range crystalline orderings may compete and cause crystallization failure. Recent developments indicate that this competition results in a higher structural difference between the liquid phase and any possible crystal phase, and manifests in a larger interfacial penalty between those phases^23,24^.

Our work draws inspiration from these latter studies: we systematically investigate structural competition between different types of crystalline ordering in a full two-dimensional landscape of related systems, and provide a link, for multiple glass-formers in a unified manner, between vitrification and the existence of stable crystal polymorphs nearby in alchemical space. Specifically, we show that glass-forming fluids of hard polyhedral shapes contain local structures that are favored in crystals formed entropically from particles of slightly altered shape; that is, from neighboring shapes in ‘shape space’^25,26^. Rather than arrange into a crystal, particles self-organize due to directional entropic forces^27,28^ into two or more local motifs that are accessible and thermodynamically preferred in crystallizing systems comprised of particles that are nearby in shape space. These motifs exist in each glass-forming fluid at ratios that prevent crystallization into any one crystal structure. This local structural competition creates an ‘identity crisis’ in the fluid and promotes vitrification.

## Results

### The self-assembly landscape

**Fig. 1:**
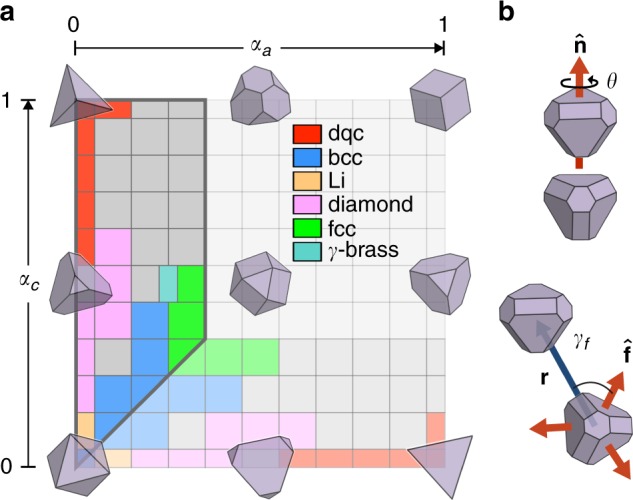
Shape space and motif analysis methods. **a** The spheric triangle invariant 323 family, with the portion of the shape space explored in this study outlined. The remaining region is colored a light gray; for details on self-assembly behavior in this region, see Damasceno et al.^27^ and Klotsa et al.^29^ Sample particle shapes are overlaid above corresponding regions of shape space, and regions are colored according to the assembled structure of the corresponding particle shape at densities between *ϕ* = 0.48 and *ϕ* = 0.64. At (*α*_*a*_, *α*_*c*_) = (0, 0.2), the system assembles into a compressed derivative of diamond with lower symmetry, but that region is colored identically to the other (cubic) diamond-formers to emphasize the similarity of these structures. At (*α*_*a*_, *α*_*c*_) = (0.3, 0.3), assembly into *bcc* occurs at *ϕ* = 0.64, while assembly into *fcc* occurs at lower densities; we color this region by the structure it assembles at the lowest density. For a broad swath of the highlighted landscape, colored gray, assembly fails to occur at any investigated density. **b** Characterization of local pairwise motifs. *θ* denotes the minimal angle associated with the rotation (about $\widehat {\mathbf{n}}$) that orients a particle identically to its nearest neighbor. *γ* denotes the minimal angle associated with the projection of **r** onto the set of unit vectors pointing to some feature of the non-truncated version of the particle shape. In this example, vectors point to centers of the faces of the non-truncated particle shape, and *γ*_*f*_ is the angle associated with the projection onto $\widehat {\mathbf{f}}$

Figure 1a shows results of hard particle Monte Carlo (HPMC) simulations of model glass and crystal-formers comprised of hard polyhedra contained in the spheric triangle invariant 323 family^25^, a set of convex polyhedra formed by truncating the vertices and edges of a tetrahedron by sets of planes at varying radial distances from the polyhedron center. The two-dimensional 323 family of polyhedra allows us to investigate shape perturbations in a tractable manner, since the more general space of all possible particle shapes is infinite-dimensional. We use a convention employed previously^30^ and define truncation parameters *α*_*a*_ and *α*_*c*_ such that the corners of the shape space are formed by (*α*_*a*_, *α*_*c*_) = (1, 1), denoting a cube, (*α*_*a*_, *α*_*c*_) = (0, 0), denoting an octahedron, and (*α*_*a*_, *α*_*c*_) = (0, 1) and (1, 0), both denoting a tetrahedron. This family is identical under reflection across the line *α*_*a*_ = *α*_*c*_. It was discovered previously^29^ that systems in certain regions of this shape space assemble into a rich variety of colloidal crystals. Particles within this family with large tetrahedrally-coordinated facets and smaller facets due to edge or vertex truncation self-assemble into a dodecagonal quasicrystal (dqc)^27,29,31^. With increasing truncation, eventually a region of shape space is reached where cubic diamond or a lower-symmetry diamond derivative is stabilized^27,29^. Close to the diagonal of the shape family, where particles possess octahedral symmetry, body-centered cubic and face-centered cubic structures assemble, with the exception of a region of shape space for which the complex high-pressure lithium structure (*cI*16-Li) is often observed^27,29,30,32^. More complicated *γ*-brass (*cI*52-Cu_5_Zn_8_), *β*-Mn (*cP*20-Mn), and BC8 silicon (*cI*16-Si) structures are also observed to assemble from shapes in select, narrow regions of this shape space^29^. Systems comprised of particles in other regions of shape space remain disordered at densities ranging from *ϕ* = 0.50 to 0.65^29^. We independently reproduced these findings for {0 ≤ *α*_*a*_ ≤ 0.3, 0 ≤ *α*_*c*_ ≤ 1} at a shape space grid resolution of Δ*α* = 0.1, finding the assembly of the *γ*-brass structure at finer resolution at (*α*_*a*_, *α*_*c*_) = (0.25, 0.5). Supplementary Fig. 9 shows the lowest packing fraction at which crystallization was observed for every crystallizing state point. Crystallization times at the lowest packing fraction for self-assembly are also shown.

Our focus here is on two systems, at (*α*_*a*_, *α*_*c*_) = (0, 0.5) and (*α*_*a*_, *α*_*c*_) = (0.2, 0.5), that are flanked by crystal-formers in shape space. These systems fail to crystallize despite excessively long simulation runs, and in fact display all of the usual characteristic dynamics of glass formers^33,34^, including systems of hard tetrahedra, octahedra, and triangular cupolae^35^, as we will describe in the next section. We hypothesize that multiple locally ordered motifs arising in these glass-formers are present and dominant in crystals formed by nearby shapes, and that these motifs exist simultaneously in the glass-formers at ratios that promote vitrification by preventing crystallization into any one crystal at all investigated densities. We assert that these motifs are accessible to the glass-former at intermediate densities because they are easily sampled by shapes nearby in shape space.

### Dynamical signatures of glass formation

**Fig. 2:**
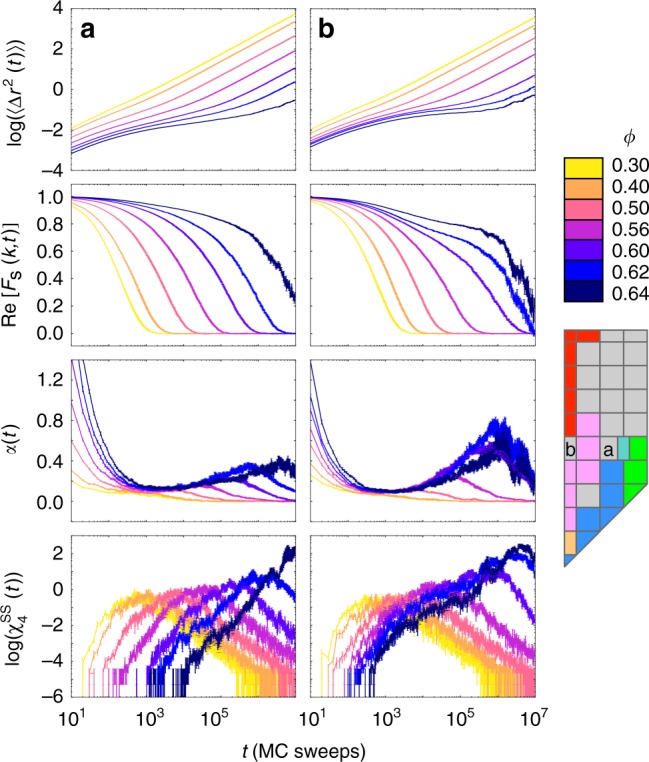
Dynamical signatures of glass formation in two example systems. Panels show the mean-squared displacement 〈Δ*r*^2^(*t*)〉, the real part of the self-intermediate scattering function *F*_s_(*k*, *t*), the non-Gaussian parameter *α*(*t*), and the self-part of the four-point susceptibility $\chi _4^{{\mathrm{SS}}}(t)$, measured at a variety of densities for two disordered state points at (*α*_*a*_, *α*_*c*_) = (**a**) (0.2, 0.5) and (**b**) (0, 0.5). Signatures in all four order parameters indicate that these systems are glass-formers. The increase in *α*(*t*) as *t* goes to zero is due to the discrete nature of Monte Carlo sampling; see the Supplementary Discussion for more detail. Error bars are calculated in a manner detailed in the Supplementary Methods

The glass-forming behavior of the systems (*α*_*a*_, *α*_*c*_) = (0, 0.5) and (*α*_*a*_, *α*_*c*_) = (0.2, 0.5) is summarized in Fig. 2. We report plateaus in the mean-squared displacement 〈Δ*r*^2^(*t*)〉 and the real part of the self-intermediate scattering function *F*_s_(*k*,*t*), which indicate caging in our systems, and peaks in the non-Gaussian parameter *α*(*t*)^36,37^ and the self-part of the four-point susceptibility $\chi _4^{{\mathrm{SS}}}(t)$^38,39^, which indicate dynamical heterogeneity associated with relaxation events. (These dynamical signatures are explicitly defined and detailed further in the Supplementary Methods.) Thus, we find that our systems display canonical behavior associated with glass formation. One notable difference between our system and other glass-forming models simulated via molecular dynamics appears in the non-Gaussian parameter: for systems simulated via molecular dynamics, *α* goes to zero as *t* goes to zero because the system is Gaussian at short times. As expected for a Monte Carlo simulation, however, we find that *α* does not go to zero at short times, and instead increases as *t* decreases in the short time regime. This behavior is due to the discrete nature of particle moves during Monte Carlo sampling. As *t* goes to zero our probability distribution of particle positions can be thought of as that of a random walk in which just one step is attempted, and a back-of-the-envelope calculation of *α* in an associated toy model gives values that are comparable to those we see at short times in our systems. See the Supplementary Discussion for more detail.

### Local structure in glass-forming fluids

**Fig. 3:**
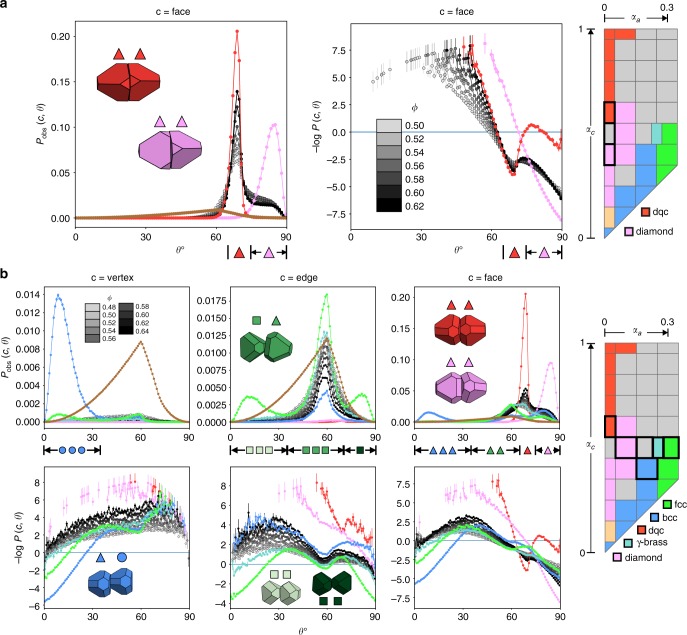
Competition of structural motifs in two example glass-formers. Competing pairwise motifs dominate in ordered structures self-assembled from shapes nearby in shape space. Panels show probabilities of observing certain pairwise configurations, *P*_obs_(*c*, *θ*), and the negative log of the normalized distributions, −log*P*(*c*, *θ*), for disordered systems at the indicated densities and nearby crystals at *ϕ* = 0.62 (or *ϕ* = 0.6 for *γ*-brass). Error bars are calculated in a manner detailed in the Motif identification section of the Methods. **a** Competition between face-connected aligned and twisted motifs at (*α*_*a*_, *α*_*c*_) = (0, 0.5). Motifs are prevalent in nearby diamond and dodecagonal quasicrystal (dqc) structures. **b** Competition between face-connected aligned and twisted motifs and a face-edge connected motif at (*α*_*a*_, *α*_*c*_) = (0.2, 0.5). Motifs are prevalent in nearby diamond, dqc, *fcc*, and *γ*-brass structures

Figure 3 displays the local structural motifs we observe for the glass-forming systems at (*α*_*a*_, *α*_*c*_) = (**a**) (0, 0.5) and (**b**) (0.2, 0.5) at a variety of densities, as well as motifs observed in crystals nearby in shape space at *ϕ* = 0.62 and *ϕ* = 0.6. (Supplementary Fig. 10 shows snapshots of these systems and others considered in this paper.) We define motifs as pairwise configurations of each particle and its nearest neighbor, and classify them by their connection type (face, edge or vertex) and relative particle misorientation *θ* as detailed in the Methods section and shown in Fig. 1b. Our analysis reveals that every competing motif in the investigated glass-formers is characteristic of a nearby ordered structure. These characteristic motifs compete in each disordered fluid at stoichiometries that impede crystallization into any one particular crystal structure.

Panels show probabilities of observing certain pairwise configurations, *P*_obs_(*c*, *θ*), and negative logs of the distributions normalized with respect to an ‘ideal gas’ of non-interacting particles of the same symmetry group, −log*P*(*c*, *θ*). The brown curves indicate *P*_rand_(*c*, *θ*), the connection type and misorientation distribution for the ideal gas, and other curves are colored according to their location in shape space. Motifs that are characteristic of nearby crystal structures and exist in significant number in each glass-forming fluid are shown in insets in Fig. 3a and the top row of panels in Fig. 3b, while motifs that are characteristic of nearby crystal structures and do not exist in significant number in the glass-forming fluid at (*α*_*a*_, *α*_*c*_) = (0.2, 0.5) are shown in insets in the bottom row of panels in Fig. 3b. Ranges of *θ* that characterize motifs are shown as small black bars, with symbols between them that represent the motif. The symbols are colored according to the crystals in which each motif is dominant. Circles indicate vertex-connection, squares indicate edge-connection, and triangles indicate face-connection. Heterogeneous connections are possible, where one member of the pair has one connection type, and the other has another connection type.

Figure 3a shows the glass-former at the location (*α*_*a*_, *α*_*c*_) = (0, 0.5) in shape space, sandwiched between shapes that form the diamond structure and shapes that form a dodecagonal quasicrystal (dqc). We find that the glass-former is increasingly dominated by face-connected particles as density increases. Vertex connection is heavily suppressed, even at lower densities around *ϕ* = 0.5, and edge connection is increasingly suppressed with increasing density. Statistics associated with all connection types are shown in Supplementary Fig. 11; here we focus on the case of face connection. The function (−log*P*(*f*, *θ*)) for the disordered system shows two distinct basins, around *θ* = 90° and *θ* = 70°, and the depth of both basins increases with density. The nearby dodecagonal quasicrystal shows a corresponding basin around *θ* = 70°, while the nearby diamond structure shows a basin at *θ* = 90°. By inspection, the basin around *θ* = 70° corresponds to an ‘aligned’ motif (drawn in red) consisting of two particles face-to-face and rotated such that their truncated vertices are aligned; a perfectly-constructed pair with this configuration has a misorientation θ ~ 70.53°. The basin at *θ* = 90°, by contrast, corresponds to a ‘twisted’ motif (drawn in pink) consisting of two particles face-to-face and twisted such that the edge midpoints of one particle align with the truncated vertices of the other. These motifs coexist in the glass-forming fluid, and each motif is dominant in a nearby crystal: the aligned motif is abundant in the nearby dodecagonal quasicrystal and absent in the nearby diamond structure, while the twisted motif is abundant in the nearby diamond structure and absent in the nearby quasicrystal. We will show that these motifs compete in the glass-forming fluid by existing at ratios that prevent crystallization into either structure.

Figure 3b shows results for the second example glass-forming shape, located at (*α*_*a*_, *α*_*c*_) = (0.2, 0.5) and surrounded in shape space by shapes that self-assemble into a dodecagonal quasicrystal, the diamond crystal, a *bcc* crystal, an *fcc* crystal, and a *γ*-brass crystal structure. This competition is more complicated, due to multiple nearby crystal structures and the fact that some nearby crystal structures are characterized by multiple pairwise motifs. Each crystal structure, however, does have particular pairwise configurations that are more probable for that structure than any other structure and more probable than in the ideal gas; we will take these as the motifs that are characteristic of each crystal structure.

We find that vertex-connection is heavily suppressed in the glass-forming system at all investigated densities. This connection type is characteristic of the nearby *bcc* crystal; more specifically, the *bcc* crystal is characterized by the pairwise motif (drawn in blue) consisting of two particles with a face-vertex connection and a misorientation *θ* = 0°. Regarding edge-connection, the disordered system has a local basin in −log*P*(*e*, *θ*) around 58° that persists at all densities, although the number of edge-connections in the disordered system decreases as density increases. This basin is characteristic of the nearby *fcc* crystal, and corresponds by inspection to the pairwise motif drawn in green, consisting of an edge-face connection in which the edge of one particle bisects the face of its nearest neighbor. A perfectly-constructed pair with this configuration has misorientation *θ* ~ 54.74°. (The *fcc* structure also shows basins in −log*P*(*e*, *θ*) around *θ* = 0° and *θ* = 90°. By inspection, these basins correspond to the pairwise configurations drawn in dark green and light green. They do not appear with any significance in the disordered fluid at any density, however.) Regarding face-connection, the disordered system shows a basin in −log*P*(*f*, *θ*) around 58°, which becomes less significant as density increases, and basins around 70° and 90°, which become more significant as density increases. The basin around 58° corresponds to the other half of the aforementioned face-edge connected motif that is characteristic of *fcc* and drawn in green. The basin around 70° corresponds to the face-connected aligned pairwise configuration, drawn in red, that is characteristic of the nearby dodecagonal quasicrystal. The basin around 90° corresponds to the face-connected twisted pairwise configuration, drawn in pink, that is characteristic of the nearby diamond structure. Thus, motifs that are characteristic of nearby crystal structures are shown to coexist in the disordered fluid at all investigated densities.

### Structural competition and identity crisis

**Fig. 4:**
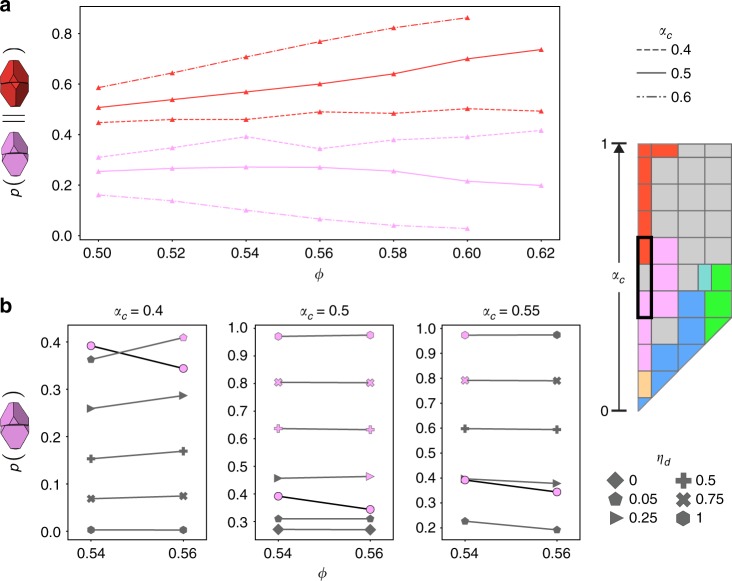
Tuning of local structure in disordered and crystal-forming fluids. Disordered and crystal-forming fluids are structurally different, and tuning their fluid structure can promote or suppress crystallization. Systems are identified by *α*_*c*_, indicating their location in shape space at (*α*_*a*_, *α*_*c*_) = (0, *α*_*c*_), and the relevant location in shape space is outlined in black in the image to the right of the plots. **a** Twisted and aligned motif fractions, or probabilities of observing either motif, as a function of packing fraction for the disordered system at (*α*_*a*_, *α*_*c*_) = (0, 0.5) and nearby crystal-formers. Pink triangles represent twisted motif fractions, and red triangles represent aligned motif fractions. Different line styles indicate different locations in shape space. Crystal-forming fluids contain higher fractions of the motifs that dominate in the assembled structures. **b** Doping via the introduction of rigid local structural motifs into dense fluids influences assembly behavior, causing crystallization for systems that might otherwise vitrify, and vitrification for systems that otherwise crystallize. Panels show twisted motif fraction, or the probability of observing the twisted motif, for (pre-cursor) fluids during doping experiments at different locations in shape space. Symbols indicate different dopant fractions *η*_*d*_. The dopant dimer is the twisted motif for *α*_*c*_ = 0.5 and *α*_*c*_ = 0.55, in which case doping promotes self-assembly into the diamond structure. For *α*_*c*_ = 0.4, the dopant dimer is the aligned motif, in which case doping disrupts self-assembly into the diamond structure. Symbols are colored pink if the system self-assembles into diamond on the time scales of our simulations. The threshold for assembly established by the undoped system at (*α*_*a*_, *α*_*c*_) = (0, 0.4) is indicated by circles connected by a black line. In all panels, error bars are smaller than the symbols, and are calculated in a manner detailed in the Precursor fluid identification section of the Methods

We next demonstrate that the motifs found in the glass-forming fluid at (*α*_*a*_, *α*_*c*_) = (0, 0.5) coexist at ratios that hinder assembly into any crystal structure. We first consider the structural difference between the glass-forming fluid at (*α*_*a*_, *α*_*c*_) = (0, 0.5) and the nearby (pre-cursor) crystal-forming fluids at (*α*_*a*_, *α*_*c*_) = (0, 0.4) and (*α*_*a*_, *α*_*c*_) = (0, 0.6). Figure 4a shows the fraction of particles in the face-connected twisted motif (shown as pink triangles) and face-connected aligned motif (shown as red triangles) as a function of density for the glass-forming fluid at (*α*_*a*_, *α*_*c*_) = (0, 0.5) and the nearby (pre-cursor) crystal-forming fluids at (*α*_*a*_, *α*_*c*_) = (0, 0.4) and (*α*_*a*_, *α*_*c*_) = (0, 0.6). The fluid at (*α*_*a*_, *α*_*c*_) = (0, 0.4) coexists with the diamond structure at *ϕ* = 0.54, and assembles solely the diamond structure at 0.56 ≤ *ϕ* ≤ 0.62. The fluid at (*α*_*a*_, *α*_*c*_) = (0, 0.6) assembles into the dodecagonal quasicrystal at *ϕ* = 0.6; shown here is a trajectory at the same state point that did not assemble into the quasicrystal on the time scale of our simulation, but for which we collected ample data in the fluid regime. We believe that, at long enough times, the system shown here would assemble into the quasicrystal, since assembly was observed in a system that differed from this one only by its random initial conditions, the assembled quasicrystal was found to be stable at densities as low as *ϕ* = 0.56 according to melting studies detailed later in this paper and in the Supplementary Discussion, and the assembled quasicrystal has a motif stoichiometry that is very similar to the fluid one shown here. Supplementary Fig. 12 compares motif stoichiometry and system pressure for the fluid shown here and the assembled quasicrystal at this state point.

At densities relevant to crystallization, the glass-forming fluid contains fewer twisted motifs (associated with the diamond structure) than the nearby fluid that assembles into diamond, and fewer aligned motifs (associated with the dodecagonal quasicrystal) than the nearby fluid that assembles into the quasicrystal. Evidently, the fraction of particles forming twisted motifs in the pre-cursor diamond-forming fluid (~0.34–0.42) for 0.54 ≤ *ϕ* ≤ 0.62 is high enough to drive crystallization into diamond, and the fraction of particles forming aligned motifs in the pre-cursor quasicrystal-forming fluid (~0.86) at *ϕ* = 0.6 is high enough to drive self-assembly into the dodecagonal quasicrystal. By contrast, the glass-forming fluid exhibits fractions of particles forming the twisted motif in the range (~0.20–0.27) and fractions of particles forming the aligned motif in the range (~0.51–0.74) at all investigated densities, preventing either crystal from forming. (Supplementary Fig. 2 in the Supplementary Discussion contains plots of every motif in these fluids as a function of packing fraction, as well as motif fractions for the assembled structures.)

We verified that the twisted motif fraction shown in the pre-cursor fluid of the diamond-former was necessary for crystallization into diamond via a set of ‘doping simulations’ in which we artificially inserted the face-connected aligned motif (associated with the quasicrystal) into the diamond-forming fluid at (*α*_*a*_, *α*_*c*_) = (0, 0.4), and the face-connected twisted motif (of the diamond crystal) into disordered fluids at (0, 0.5) and (0, 0.55). For these simulations, we rigidly connected a fraction *η*_*d*_ of particles in each dense fluid into pairs to form the relevant dimer motifs, and ran simulations at densities *ϕ* = 0.54 and *ϕ* = 0.56 for *η*_*d*_ ranging from 0.05 to 1.0. Via this mechanism, we were able to either artificially enhance or suppress the fraction of particles forming twisted pairwise motifs, and observe consequent assembly or non-assembly behavior. Our results are summarized in Fig. 4b, which shows twisted motif fractions as a function of packing fraction for (pre-cursor) fluids of doped and undoped systems. Symbols are colored pink if the system self-assembled into diamond on the time scale of our simulation at that state point. Pink symbols only appear at twisted motif fractions above the threshold established by the diamond-forming undoped system at (*α*_*a*_, *α*_*c*_) = (0, 0.4), indicated by circles connected by a black line, for all investigated locations in shape space and doping schemes. At the point in shape space (*α*_*a*_, *α*_*c*_) = (0, 0.4), introduction of the aligned motif of the quasicrystal caused assembly failure in the would-be diamond-former when *η*_*d*_ ≥ 0.25. For these crystallization-thwarting doping schemes, the fraction of particles in the twisted motif is observed to be below the threshold shown by the diamond-forming undoped system. At (*α*_*a*_, *α*_*c*_) = (0, 0.5) and (*α*_*a*_, *α*_*c*_) = (0, 0.55), introduction of the twisted motif of diamond to the disordered fluids caused crystallization into diamond at *η*_*d*_ ≥ 0.25 and *η*_*d*_ ≥ 0.75, respectively. For these crystallization-inducing doping schemes, the fraction of particles in the twisted motif is observed to be above the threshold established by the diamond-forming undoped system at (*α*_*a*_, *α*_*c*_) = (0, 0.4). Previous studies have additionally shown that systems composed entirely of aligned motifs made of non-truncated tetrahedra^40^ and tetrahedra with a slightly modified vertex truncation^41^ assemble the dodecagonal quasicrystal at long times under various simulation strategies. This provides some evidence that the aligned motif is capable of promoting self-assembly into the dodecagonal quasicrystal. Our results demonstrate clearly that the competition between the high fractions of face-connected twisted and aligned motifs in the glass-forming fluid at (*α*_*a*_, *α*_*c*_) = (0, 0.5) is responsible for its failure to crystallize, since this competition can be artificially tuned to promote self-assembly in systems that may otherwise vitrify, or suppress self-assembly in systems that may otherwise crystallize. Supplementary Fig. 13 shows a phase diagram summarizing the results of all doping simulations, Supplementary Fig. 14 displays example trajectories of doped systems at (*α*_*a*_, *α*_*c*_, *ϕ*) = (0, 0.5, 0.56), and Supplementary Fig. 15 displays example trajectories of doped systems at (*α*_*a*_, *α*_*c*_, *ϕ*) = (0, 0.4, 0.56).

We also attempted to dope systems near (*α*_*a*_, *α*_*c*_) = (0, 0.5) with the aligned motif, to coax them into forming the dodecagonal quasicrystal, and to dope systems at (*α*_*a*_, *α*_*c*_) = (0.2, 0.5) with motifs dominant in nearby *bcc*, *fcc*, and diamond structures, to coax them into forming those crystals. However, we were unsuccessful in those attempts, indicating perhaps that appropriate local structure is a necessary but not sufficient condition for crystallization, at least on the time and size scales of our simulations.

Supplementary Fig. 3 in the Supplementary Discussion shows the structural difference between the glass-forming fluid at (*α*_*a*_, *α*_*c*_) = (0.2, 0.5) and the nearby pre-cursor crystal-forming fluids at (*α*_*a*_, *α*_*c*_) = (0.1, 0.5), (0.2, 0.4), (0.25, 0.5), and (0.3, 0.5). It portrays a similar phenomenon to that of Fig. 4a: the glass-former is structurally distinct from each nearby crystal-former, containing fewer motifs associated with any crystal structure than the nearby fluid that assembles that structure at densities relevant to crystallization. Supplementary Figs. 4 and 5 in the Supplementary Discussion show motif fraction in pre-cursor or disordered fluids across the entire shape landscape at two example densities (*ϕ* = 0.54 and 0.6 respectively), demonstrating the general trend that motifs tend to be more abundant in regions of shape space in which fluids self-assemble into the crystals associated with those motifs. Glass-forming fluids lie approximately between these regions, and thus contain significant motif fractions corresponding to multiple crystals. This is the origin of the ‘identity crisis’.

### Crystal stability tests

Stability tests for candidate crystal structures in regions near the glass-formers at (*α*_*a*_, *α*_*c*_) = (0, 0.5) and (0.2, 0.5) provide further proof that a local structural identity crisis in the dense fluid is responsible for vitrification. We systematically changed the shape of particles comprising crystal structures near these glass-formers in shape space, transforming the particle shape incrementally into the glass-forming shape, and measured melting density and pressure as a function of particle shape. Procedural details are contained in the Supplementary Methods. Our results show that at each investigated glass-forming location in shape space, select crystals remain stable in density regimes for which we observed no crystallization from the fluid. This strongly suggests that these glass-forming fluids are ‘super-cooled,’ or more accurately, super-compressed. Supplementary Figs. 6 and 7 in the Supplementary Discussion summarize our results, and show plots of melting density as a function of particle shape for several candidate crystal structures. These melting lines are in the spirit of other phase diagrams calculated as functions of various system control parameters^24,42^. In those cases, it was observed that good glass-formers appear near eutectic points in these phase diagrams, when the stable crystal structure undergoes a cross-over. We find evidence of eutectic points near our glass-forming state points, although at each glass-forming location in shape space, there is a crystal structure that is more stable than the others investigated and whose stability easily extends into the fluid density regime. The glass-former at (*α*_*a*_, *α*_*c*_) = (0, 0.5), in particular, appears to be at a location in shape space for which the nearby diamond crystal is actually more stable than in the region for which diamond self-assembles. Thus, we argue that a close examination of the fluid phase itself, and especially its structural make-up, is necessary for a complete understanding of crystallization failure in these systems.

### Alchemical Monte Carlo

**Fig. 5:**
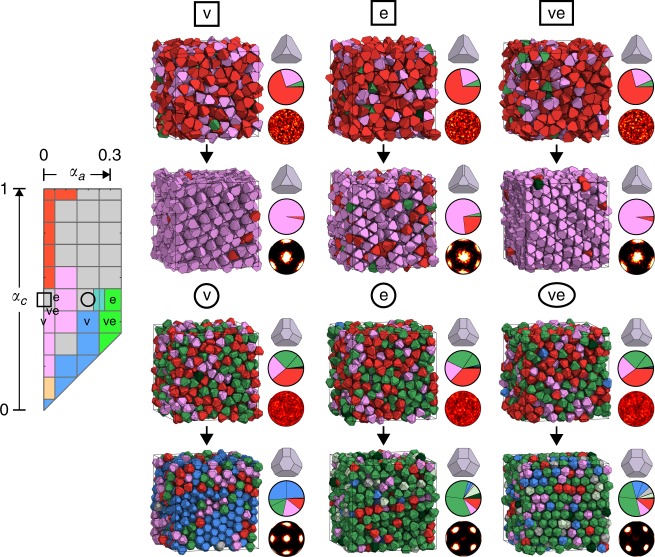
Alchemical Monte Carlo simulations of would-be glass-formers. Would-be glass-formers escape their identity crisis and crystallize when allowed to explore their surrounding shape space via alchemical Monte Carlo simulation. Squares indicate simulations at the glass-forming state point (*α*_*a*_, *α*_*c*_) = (0, 0.5), while circles correspond to simulations at (*α*_*a*_, *α*_*c*_) = (0.2, 0.5). Empty symbols overlaid above the shape space indicate system position at the start of Alch-MC sampling, and letters indicate system position after 20–30 million MC sweeps of vertex truncation (v), edge truncation (e), or both vertex and edge truncation (ve) sampling. System snapshots, particle shapes, pie charts of pairwise motif fractions, and bond-order diagrams^31^ are shown for initial and final frames of each Alch-MC simulation. Pie chart wedges are colored according to the motifs identified in Fig. 3. Wedges colored gray represent (connection type, *θ*) regimes that were not identified with any crystal structure. Pie chart wedges colored identically represent motifs characteristic of the same crystal structure that differ only by connection type. In those cases, the motif with face connection is always drawn second, proceeding in a counter-clockwise fashion. The hexagonal bond-order diagram resulting from edge Alch-MC sampling at (*α*_*a*_, *α*_*c*_) = (0, 0.5) is a consequence of wurtzite-like structural motifs due to the presence of stacking faults in the system. Crystalline structures resulting from edge and vertex-edge Alch-MC sampling at (*α*_*a*_, *α*_*c*_) = (0.2, 0.5) contain multiple grains and stacking faults; associated bond-order diagrams show the local environment of particles in just a single grain. In all cases shown, disordered dense fluids avoid vitrification and instead form crystals dominated by a single pairwise motif

We provide additional evidence that an identity crisis in alchemical space promotes glass formation in hard particle fluids by allowing disordered systems to explore their surrounding shape space through alchemical Monte Carlo (Alch-MC) sampling^26^. In this technique, particle shape (defined in this case by the truncation parameters *α*_*a*_ and *α*_*c*_) is treated as a thermodynamic variable, and allowed to fluctuate in a generalized thermodynamic ensemble at constant (zero) conjugate alchemical potential. We sampled disordered systems at (*α*_*a*_, *α*_*c*_) = (0, 0.5) and (0.2, 0.5) via Alch-MC at a range of densities between *ϕ* = 0.52 and *ϕ* = 0.64. At each density, we ran simulations in which we allowed only the vertex truncation parameter *α*_*c*_, only the edge truncation parameter *α*_*a*_, or both to fluctuate. We constrained systems to only explore the area inside a square of side length Δ*α* = 0.2 centered at their initial position in shape space by imposing appropriate limits on each *α* parameter during sampling. In each simulation, all particle shapes were identical and sampled simultaneously. Figure 5 shows results for alchemical sampling in both example glass-forming systems. All simulations shown are at *ϕ* = 0.62, except the case of edge truncation sampling at (*α*_*a*_, *α*_*c*_) = (0.2, 0.5), which is shown at *ϕ* = 0.60. Instead of forming a glass, each disordered system crystallizes into a ‘nearby’ ordered structure by slightly altering its particle shape and accordingly adopting a larger fraction of the associated crystalline pairwise motif. Thus we see that, given the thermodynamic choice, these hard particle fluids escape schizophrenic regions of shape space, and assemble into nearby crystalline structures typically dominated by one motif. Results for Alch-MC simulations at all investigated densities are included in Supplementary Figs. 16–21.

### An additional shape space

Finally, we show that our identity crisis hypothesis is independent of particle symmetry and adjacent crystal structure by investigating another glass-forming system in a different shape space, defined by the spheric triangle invariant 423 family^25,29^. This glass-former consists of hard particles with octahedral symmetry, located in a shape space region surrounded by shapes that form either *bcc* or a high-pressure lithium phase that is likely metastable to *bcc*^29^. We observe two competing motifs in this glass-former, each dominant in the nearby *bcc* or metastable high-pressure lithium structures. We allowed the glass-former to explore its immediate surroundings in shape space through Alch-MC sampling, and found that it consequently escaped its identity crisis by adopting a nearby particle shape that forms *bcc*. More detail is provided in the Supplementary Discussion, and our results are summarized in Supplementary Fig. 8.

## Discussion

Our results show that the concept of alchemical (here, shape) space is a useful lens through which to understand the vitrification of hard particle fluids. Crystallization fails in these systems due to the presence of multiple local structures, each of which is preferred in crystals formed by particles nearby in shape space. These structures compete by existing at ratios in the glass-formers that impede crystallization into any one crystal. Thus the entropic colloidal glass transition is caused by an identity crisis in shape space in which the glass-formers are unable to settle on any one particular set of local motifs consistent with a single crystal structure. In relation to other studies of local structure in glassy liquids, our findings most closely align with the results of Tanaka et al.^18,24,43^, who posit that multiple types of ordering compete and suppress crystallization via the literal suppression of crystalline pre-cursors in super-cooled liquids. ref. ^24^ is especially relevant here: in that work, coauthors found that glass-forming ability is positively correlated with increased competition between multiple types of crystalline ordering, found near eutectic points when either the size ratio of a binary hard disk system or the strength of tetrahedrality in a modified Stillinger-Weber^44^ model system is varied. Our results expand on these ideas in the context of hard-particle glass-formers: we find glass formation via multiple types of competing crystalline order on a very local level, each prevalent in nearby ordered structures in a two-dimensional alchemical landscape. Slightly modified particles have correspondingly modified preferences for assuming various local structural motifs, and thus serve as indicators of the competing preferences of the system under investigation.

The alchemical framework considered in this work may also be useful for understanding glass-formers in different contexts. Many previous studies have manipulated degrees of freedom in glass-forming systems to relieve or increase frustration. Stoichiometry in binary Lennard-Jones systems^45^, polydispersity in two^20,24^ and three^46^ dimensions, salt concentration in a water-salt mixture^47^, bias towards five-fold local ordering in two^19^ and three^48^ dimensions, bond tetrahedrality^24,42^, and even the curvature of three-dimensional space^49^ have been tuned in pursuit of turning a glass-former into a crystal-former or vice-versa. In those cases, results typically show that local structures in frustrated glass-formers are related to local structures in one or more corresponding non-frustrated crystals. Considering these degrees of freedom as alchemical parameters, and their ‘tuning’ as controlled exploration of alchemical space, may provide a useful unifying perspective.

## Methods

### Software

We used the hard particle Monte Carlo (HPMC)^50^ simulation extension of the open-source simulation toolkit HOOMD-blue^51,52^ for both fixed-shape and Alch-MC simulations. In all simulations, trial translations and orientations of particles of fixed shape were attempted, and moves were rejected if they resulted in particle overlaps. Alch-MC required additional trial shape change moves, as discussed below. The computational workflow and data management for this project was facilitated by the signac data management framework^53,54^. We also used the open-source analysis package freud^55^ to calculate fractions of particles in various crystalline environments as detailed in the Supplementary Methods.

### Assembly simulations

To explore the self-assembly behavior of particles in the spheric triangle invariant 323 family, we sampled one-component systems, in equilibrium, composed of particle shapes that satisfy {0 ≤ *α*_*a*_ ≤ 0.3, 0 ≤ *α*_*c*_ ≤ 1}. We used a grid of resolution Δ*α* = 0.1 in both *α*_*a*_ and *α*_*c*_. For each particle shape, we sampled equilibrium behavior in the isochoric ensemble over a range of densities between *ϕ* = 0.48 and *ϕ* = 0.64. Simulations of 4096 particles were run for about 100 million MC sweeps or until self-assembly was observed. Detailed simulation protocols are provided in the Supplementary Methods. Self-assembled phases were identified by eye and quantified by the bond-order diagram^31^, radial distribution function, and diffraction pattern.

### Motif identification

We identified the motif composed of particle *i* and its nearest neighbor, particle *j*, via two parameters. The first is associated with the ‘connection type’ (*c*_*ij*_, hereafter *c*) between *i* and *j*. *i* is ‘face-connected’ (*c* = *f*) to *j* if particle *i*'s face is the closest feature to the connection vector **r**_*ij*_ (from the center of *i*, **r**_*i*_, to the center of *j*, **r**_*j*_), *i* is ‘edge-connected’ (*c* = *e*) to *j* if *i*'s (truncated) edge is closest, or *i* is ‘vertex-connected’ (*c* = *v*) to *j* if *i*'s (truncated) vertex is closest to the connection vector. To calculate the connection type, we considered first the non-truncated tetrahedron *i*_tet_ located at **r**_*i*_ and oriented identically to *i*. We identified the four unit vectors $\{ \widehat {\mathbf{f}}_i\}$ that point from **r**_*i*_ to the faces of *i*_tet_, the six unit vectors $\{ \widehat {\mathbf{e}}_i\}$ that point from **r**_*i*_ to the edges of *i*_tet_, and the four unit vectors $\{ \widehat {\mathbf{v}}_i\}$ that point from **r**_*i*_ to the vertices of *i*_tet_. We then calculated ${\mathrm{cos}}\gamma _f \equiv {\mathrm{max}}(\widehat {\mathbf{r}}_{ij} \cdot \widehat {\mathbf{f}}_i)$, ${\mathrm{cos}}\gamma _e \equiv {\mathrm{max}}(\widehat {\mathbf{r}}_{ij} \cdot \widehat {\mathbf{e}}_i)$, and ${\mathrm{cos}}\gamma _v \equiv {\mathrm{max}}(\widehat {\mathbf{r}}_{ij} \cdot \widehat {\mathbf{v}}_i)$. Motifs were categorized as face-connected if *γ*_*f*_ = min(*γ*_*f*_, *γ*_*e*_, *γ*_*v*_), or edge- or vertex-connected if *γ*_*e*_ or *γ*_*v*_ are the minimum angles, respectively.

Motifs were further distinguished by their relative misorientation *θ*_*ij*_ (hereafter *θ*), the angle of rotation required to orient *j* identically to *i*. In calculating *θ*, we took particle symmetry into account: each *θ* is actually the minimum of the set of equivalent angles $\{ \tilde{\theta} \}$, found by permuting through all possible pairs of equivalent particle orientations according to the particles’ rotation group. This group is the chiral tetrahedral point group **23** for all particles studied, with the exception of those on the diagonal of the shape space, in which case it is the chiral octahedral point group **432**. Due to particle symmetry, *θ* = 90° is the maximum possible relative misorientation for all pairwise configurations. See Fig. 1b for examples of *γ*_*f*_ and *θ*.

We categorized pairwise motifs by combining the connection type *c* with the relative misorientation *θ* via a joint discrete probability distribution *P*_obs_(*c*, *θ*_*k*_), as shown in Fig. 3:

1 $$P_{{\mathrm{obs}}}(c,\theta _k) = \frac{{N_{{\mathrm{obs}}}(c,\theta _k)}}{{\mathop {\sum}\nolimits_c {\mathop {\sum}\nolimits_{k = 1}^{n_{{\mathrm{bins}}}} {N_{{\mathrm{obs}}}(c,\theta _k)} } }}$$

*N*_obs_(*c*, *θ*_*k*_) is the number of particles observed with connection type *c* and misorientation *θ* in a bin centered at *θ*_*k*_ with width Δ*θ* = 0.9°. There are *n*_bins_ such bins for each connection type.

To determine statistically significant trends in this distribution, we normalized by the equivalent joint discrete probability distribution *P*_rand_(*c*, *θ*_*k*_) for an ‘ideal gas’ of non-interacting particles of the same symmetry group. The connection type is unrelated to the misorientation for non-interacting particles, so these probabilities can be considered separately: *P*_rand_(*c*, *θ*_*k*_) = *P*_rand_(*c*)*P*_rand_(*θ*_*k*_). We computed *P*_rand_(*θ*_*k*_) for both chiral tetrahedral and chiral octahedral point groups by generating 10 million random pairs of orientations and computing the minimum rotation angle *θ* between them with respect to the associated underlying rotation group, as detailed earlier. We note that analytical tools developed by the polycrystalline materials community^56–58^ can be brought to bear on this problem, since *P*_rand_(*θ*) for any underlying particle symmetry group maps to the random grain boundary misorientation angle distribution for that same underlying (crystal grain) symmetry group. For our purposes, however, it was sufficient to numerically calculate *P*_rand_(*θ*). We computed *P*_rand_(*c*) for the chiral tetrahedral point group by generating 2.5 million pairs of particles of appropriate symmetry with random orientations and a random unit displacement vector between them. We then computed connection types for these pairs in the manner detailed above. *P*_rand_(*c*) for the chiral octahedral point group was not ultimately necessary for our analysis, but could be found in a similar manner.

We also computed the negative log of the joint probability distribution, normalized with respect to an ideal gas:

2 $$- {\mathrm{log}}\,P(c,\theta _k) = - {\mathrm{log}}\frac{{P_{{\mathrm{obs}}}(c,\theta _k)}}{{P_{{\mathrm{rand}}}(c,\theta _k)}}$$

When −log*P*(*c*, *θ*_*k*_) < 0, the combination of connection type *c* and misorientation *θ*_*k*_ is more probable than in the ideal gas. Different motifs were then identified according to *θ* ranges that corresponded to local minima, or basins, in −log*P*(*c*, *θ*_*k*_). Motifs were defined by *θ*_min_ ≤ *θ* < *θ*_max_ in all cases.

The discrete *θ*_*k*_ is labeled as the continuous *θ* in Fig. 3 for simplicity. Error bars in Fig. 3 were calculated as follows: histograms over *θ* for each connection type *c* were accumulated for 10 frames (separated by 1 million MC sweeps), then *P*_obs_(*c*, *θ*_*k*_) was computed. Ensemble averages were taken over these values of *P*_obs_(*c*, *θ*_*k*_). These averages have an associated standard deviation that is shown as vertical error bars in plots of *P*_obs_(*c*, *θ*_*k*_), and that error was propagated via a first-order Taylor series expansion of −log*P*(*c*, *θ*_*k*_), shown as vertical error bars in plots of −log*P*(*c*, *θ*_*k*_). Random distributions do not have associated error.

Motifs in ordered systems were calculated at *ϕ* = 0.62, with the exception of the *γ*-brass crystal, for which motifs were calculated at *ϕ* = 0.6. To gather statistics on motifs in relevant ordered systems at *ϕ* = 0.62, we began with already well-equilibrated, self-assembled system snapshots of *N* = 4096 particles, and sampled them in the isochoric ensemble for 100 million more MC sweeps. For several state points, we began with snapshots at lower packing fractions than *ϕ* = 0.62, because they represented cleaner samples of the ordered structures of interest that assembled on the time scales of our simulations. We compressed these systems to *ϕ* = 0.62 before acquiring statistics. In the case of *γ*-brass, motif statistics were simply collected for the last 40 million MC sweeps of the self-assembling trajectory, throughout which the crystal was fully formed.

### Precursor fluid identification

For several analyses, we investigated motifs in fluids we determined were ‘pre-cursors’ to eventual self-assembled crystalline phases. We identified pre-cursor fluids as all frames of self-assembling trajectories prior to the nucleation incubation time^59^, defined in our simulations as the first frame after which approximately all crystalline particle fractions measured over the trajectory were greater than 0.1. We measured the fraction of crystalline particles in each frame using an environment matching scheme tailored to identify local per-particle bond environments associated with the relevant assembled crystal structure. This scheme is detailed further in the Supplementary Methods. If the crystalline fraction never surpassed 0.1, the system did not crystallize, and we treated the entire trajectory as the non-crystallized fluid.

To construct Fig. 4, we ran three or four replicate simulations at each density for the (*α*_*a*_, *α*_*c*_) = (0, 0.4) undoped system, to collect more statistics in the pre-cursor fluid regime. Motif fractions were ensemble-averaged over all pre-cursor fluid frames and are shown with error bars indicating the associated standard deviation of the mean. Frames in all trajectories were written at a frequency of once per 1 million MC sweeps.

### Doping simulations

We performed ‘doping’ simulations by artificially introducing select pairwise motifs into our systems and monitoring consequent assembly or non-assembly. We used isochoric Monte Carlo sampling and treated pairwise motifs as rigid bodies. Simulations were composed of 4,096 particles and run for about 100 million MC sweeps or until the system self-assembled. Overlap checks treated each rigid body as a union of convex polyhedra, and thus trial moves of pairwise motifs were rejected if either member of the pair overlapped with any other particle or pair. We employed a compression and equilibration scheme similar to that used in the hard particle MC simulations described previously; for more detail see the Supplementary Methods.

### Alchemical Monte Carlo simulations

We utilized the Alchemical Monte Carlo (Alch-MC) sampling technique detailed in earlier work^26,60–62^ and implemented in a branch of our in-house HPMC software package^50^. We initialized and compressed systems of 1000 particles to desired volume fractions in an identical manner to that described above for traditional isochoric MC sampling. We then equilibrated each system for 10 million MC sweeps at constant volume and constant particle shape. We finally ran Alch-MC simulations of each system for 20–30 million MC sweeps, allowing the fluctuation of either all particles’ vertex truncation parameter *α*_*c*_, all particles’ edge truncation parameter *α*_*a*_, or both in an (*NVTμ*) ensemble at constant (zero) conjugate alchemical potential *μ*. Alchemical shape moves were attempted with a 25% probability after every MC sweep. In simulations in which both *α*_*a*_ and *α*_*c*_ were sampled, each truncation parameter had a 50% probability of being sampled during a shape move.

## Supplementary information

Supplementary Information

## Data Availability

All data generated and analyzed in this study are available from the corresponding author upon request.
