# Supplementary material for: Identity crisis in alchemical space drives the entropic colloidal glass transition
Source: Nat Commun. 2019 Jan 8;10:64. doi: 10.1038/s41467-018-07977-2 (PMC6325105; doi:10.1038/s41467-018-07977-2)
Supplement: Supplementary file 1 — Supplementary Information [file 41467_2018_7977_MOESM1_ESM.pdf]

Supplementary Information: Identity crisis in alchemical space drives the entropic colloidal glass transition

Erin G. Teich,[1] Greg van Anders,[1,2,3] and Sharon C. Glotzer[1,2,3,4,5]

[1] *Applied Physics Program, University of Michigan, Ann Arbor MI 48109, United States*
[2] *Department of Physics, University of Michigan, Ann Arbor MI 48109, United States*
[3] *Department of Chemical Engineering, University of Michigan, Ann Arbor MI 48109, United States*
[4] *Department of Materials Science and Engineering, University of Michigan, Ann Arbor MI 48109, United States*
[5] *Biointerfaces Institute, University of Michigan, Ann Arbor MI 48109, United States*

I. SUPPLEMENTARY METHODS

A. Simulation protocols

To verify the self-assembly behavior reported by Klotsa et al.[1] and to generate our own data, we sampled one-component systems composed of particles in the spheric triangle invariant 323 family, as detailed in the main text. Simulations of 4,096 particles were run for about 100 million MC sweeps or until self-assembly was observed. At all state points corresponding to $\alpha_a < 0.3$ and $\alpha_c > 0.4$ for which self-assembly was not observed, we also simulated smaller systems of 2,624 particles for about 70-100 million MC sweeps. At one state point, $(\alpha_a, \alpha_c, \phi) = (0, 0.6, 0.6)$, self-assembly was observed around 120 million MC sweeps. To reach each density, we initialized our system in a sparse cubic array inside a cubic box, randomized the system by running isochoric Monte Carlo sampling for 10,000 MC sweeps, and progressively rescaled box vectors by a scale factor of 0.9995 until the target density was reached. After every rescaling step, isochoric Monte Carlo sampling proceeded until all particle-particle overlaps were eliminated. During the compression process, the translational trial move size was identical to its value during equilibrium sampling, but the rotational trial move size was larger in order to facilitate the removal of overlaps during fast compressions to high densities. During equilibrium sampling, rotational and translational trial move sizes were constant across all systems studied, and were chosen to most efficiently structurally relax a typical system.

To perform the "doping simulations" detailed in the main text, we employed a similar compression and equilibration strategy, with a few differences to accommodate the larger size and aspect ratio of the dimer dopants: we thermalized the system prior to compression for 1 million MC sweeps rather than 10,000 MC sweeps, and switched rotation move size during compression to its smaller equilibration value if compression was proceeding slowly.

B. Environment matching for crystallinity determination

We determined fractions of particles in a particular crystalline environment in our simulations via an "environment matching" scheme, which we have made available as part of *freud*[2], our group's open-source analysis package. We define particle i's environment as the set of vectors $\{\vec{r}_{im}\}$, where $\vec{r}_{im}$ points from the center of particle i to the center of particle m, and m is an index over i's M nearest neighbors. We then determine whether i's environment "matches" the environment of any of its K nearest neighbors: for particle k, where k is a member of the set of the K nearest neighbors of i, we determine k's environment $\{\vec{r}_{km'}\}$, where m' loops over k's M nearest neighbors. We then attempt to find a one-to-one mapping between the vectors of i's environment and the vectors of k's environment such that $|\vec{r}_{im} - \vec{r}_{km'}| < t$ for every mapping pair (m, m') for some threshold t. If that mapping is found, i's environment matches k's environment. We cluster particles according to common environment, searching over the K nearest neighbors of every particle, and label a particle as "crystalline" if it belongs to a cluster of size $s > s^*$, where s^* is some cut-off. In almost all cases, we chose $t = 0.3r^*$, where r^* is a length scale we chose to be 1.7, a typical nearest-neighbor distance in our systems. We occasionally set $t = 0.2r^*$ for finer-grained analysis of systems that were suspected to assemble into the high-pressure Lithium structure. For tracking crystallization during alchemical Monte Carlo runs, we chose the size cut-off $s^* = 5$, while for determining crystallization time, we chose the size cut-off $s^* = 1$. Environment parameters K and M were tailored according to the crystal structure whose growth or presence we wanted to detect: for *fcc*, we set $\{K, M\} = \{12, 12\}$, for *bcc*, we set $\{K, M\} = \{14, 14\}$, for diamond, we set $\{K, M\} = \{16, 4\}$, for high-pressure Lithium, we set $\{K, M\} = \{80, 11\}$, and for γ-brass, we set $K = 200$, and found M for each particle as the number of neighbors that fell within a cut-off distance of $r = 1.7$. Then, only particles with identical values of M were tested for similarity. This was necessary due to the spread in nearest neighbor number among particles in the more complicated γ-brass structure.

C. Dynamical characterization

To establish the glass-forming ability of disordered systems in this shape space, we chose particle shapes that remain disordered on the long time scales of our simulations at the densities investigated to establish phase behavior, and re-sampled one-component systems of those shapes in a broader density range. We

used Monte Carlo methods identical to those described in detail above. We equilibrated our systems for approximately 50 million MC sweeps, and then collected dynamical data for 100 million MC sweeps.

For each system, we calculated the following order parameters at logarithmic timescales: the mean-squared displacement $\langle \Delta r^2(t) \rangle$ of all particles in the system, the self-part of the intermediate scattering function $F_s(\vec{k},t)$, computed for the k-value associated with the first peak of the static structure factor, the non-Gaussian parameter $\alpha(t)$[3], and the self-part of the four-point susceptibility $\chi_4^{SS}(t)$[4,5]. These quantities are defined as follows:

$$\langle \Delta r^2(t) \rangle \equiv \frac{1}{N} \left\langle \sum_{j=1}^{N} (\Delta \vec{r}_j(t))^2 \right\rangle \tag{1}$$

$$F_s(\vec{k},t) \equiv \frac{1}{N} \left\langle \sum_{j=1}^{N} e^{i\vec{k}\cdot\Delta\vec{r}_j(t)} \right\rangle \tag{2}$$

$$\alpha(t) = \frac{3\left\langle \Delta r^4(t) \right\rangle}{5\left\langle \Delta r^2(t) \right\rangle^2} - 1 \tag{3}$$

$$\chi_4^{SS}(t) = N\left[\left\langle Q_S^2(t)\right\rangle - \left\langle Q_S(t)\right\rangle^2\right] \tag{4}$$

$$Q_S(t) = \frac{1}{N}\sum_{j=1}^{N} H\left(a - |\Delta\vec{r}_j(t)|\right) \ .$$

In all definitions above, $\Delta\vec{r}_j(t) \equiv \vec{r}_j(t) - \vec{r}_j(0)$. In the final expression, H is the Heaviside step function: $H(x) = 1$ for $x > 0$ and 0 otherwise. a is a length-scale associated with the self-overlap of any particle in the system; in this paper we took a to be the inscribing sphere radius of the particle shape for each system. In all cases, angle brackets indicate ensemble averages. We determined that relaxation in most systems is complete by about 10 million MC sweeps; thus, we broke each 100 million MC sweep trajectory into 10 windows and took appropriate ensemble averages over these windows. Error bars were determined through either error propagation or jackknife resampling; see Supplementary Method I D for more detail.

We computed $F_s(\vec{k},t)$ at every pertinent lagtime t by averaging over computed values of $F_s(\vec{k},t)$ for 10 randomly generated vectors with magnitude k in a similar manner to that described elsewhere[6]. We did this to speed up our calculations, as $F_s(\vec{k},t) = F_s(k,t)$ in an isotropic medium. We computed $F_s(\vec{k},t)$ for the k value corresponding approximately to the location of the first peak of the static structure factor of each system.

Supplementary Fig. 1 shows the static structure factors $S(k)$ for the disordered systems $(\alpha_a, \alpha_c) = (0, 0.5)$ and $(\alpha_a, \alpha_c) = (0.2, 0.5)$. We found $S(\vec{k}) \equiv \frac{1}{N}\langle \sum_{j,l=1}^{N} e^{i\vec{k}\cdot(\vec{r}_l - \vec{r}_j)}\rangle$ via the squared FFT of the number density $\rho(\vec{r})$ of the system, Gaussian-blurred for smoothness, since $S(\vec{k}) = \frac{1}{N}\left|\langle \int d\vec{r}\rho(\vec{r})e^{i\vec{k}\cdot\vec{r}}\rangle\right|^2$. We then found $S(k)$ by assuming that the system is isotropic, and spherically averaging $S(\vec{k})$ using a channel-sharing method[7]. The static structure factors we show here were calculated for the first frame of each trajectory only. They are given as functions of $k\sigma$, where σ is a length scale that characterizes the particle size of each system: $\sigma^3 = v_p$, where v_p is the particle volume (1 in all cases).

In glass-forming systems generally, $\langle \Delta r^2(t) \rangle$ and $F_s(\vec{k},t)$ increasingly display three regimes as density increases or temperature decreases: a regime at short timescales in which particles move without colliding with any others, a caging regime at intermediate timescales in which particles are caged by their neighbors and relaxation slows, and a regime at long timescales in which particles escape the confines of their cages and eventually diffuse through the system. $\alpha(t)$ gives a measure of the degree to which the distribution of particle displacements in the system deviates from a Gaussian distribution. It typically has a peak in glass-forming systems at times of large dynamical heterogeneity[8,9], when some particle motions are cooperative and therefore a subset of particle displacements is higher than that given by the expected Gaussian distribution. $\chi_4^{SS}(t)$ gives a direct measure of the dynamical heterogeneity of the system, as it is the scaled variance of the 2-point self-correlation function $Q_S(t)$: χ_4 grows from zero as heterogeneity in the dynamics of the system increases over a time window t, and decreases back to zero at long times in the dense fluid.

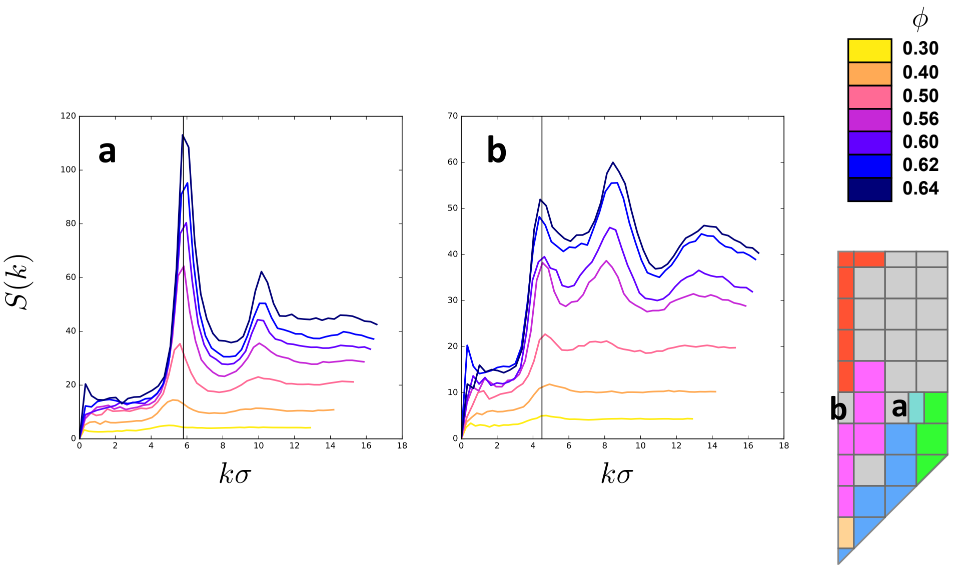

Supplementary Figure. 1: Static structure factors for the systems identified by letters in shape space. Vertical lines through each plot indicate the position of the first peak, used for calculation of the self-intermediate scattering function in the main text. These positions are $k\sigma = 5.8, 4.5$ for $(\alpha_a, \alpha_c) = (0.2, 0.5), (0, 0.5)$ respectively.

D. Error determination of dynamical order parameters

We determined error bars for each dynamical order parameter with a variety of techniques. We detail them here.

In practice we calculated $\langle \Delta r^2(t) \rangle$ in the following manner:

$$\langle \Delta r^2(t) \rangle = \frac{1}{M} \sum_{m=1}^{M} \frac{1}{N} \sum_{j=1}^{N} (\vec{r}_{jm}(t) - \vec{r}_{jm}(0))^2 \tag{5}$$

where $\vec{r}_{jm}(t)$ is the position of particle j at time t in frame m, and there are M total frames making up the ensemble average. Then we computed the variance of this average as

$$\sigma^2(t) = \frac{1}{M^2} \sum_{m=1}^{M} \sigma_m^2(t) \tag{6}$$

where $\sigma_m^2(t)$ is the variance of the mean squared displacement taken across all particles in frame m. This is error propagation via a Taylor series expansion of the total error, where we assume that measurements taken in each frame m are independent of each other. This is a reasonable assumption, since we took care to ensemble average over frames separated by times greater than the autocorrelation time.

We determined error associated with the self-part of the intermediate scattering function in a similar manner:

$$F_s(\vec{k}, t) = \frac{1}{M} \sum_{m=1}^{M} \frac{1}{L} \sum_{l=1}^{L} \frac{1}{N} \sum_{j=1}^{N} e^{i\vec{k}_l \cdot [\vec{r}_{jm}(t) - \vec{r}_{jm}(0)]} \tag{7}$$

where $\vec{k}_l$ is a point randomly chosen on the surface of a sphere of radius k via the Marsaglia[10] method during iteration l, and there are L total iterations.

Then the variance, again by Taylor series error propagation, is:

$$\sigma^2(t) = \frac{1}{M^2} \sum_{m=1}^{M} \sigma_m^2(t) \ , \tag{8}$$

where $\sigma_m^2(t)$ is the variance of the mean self-intermediate scattering function taken across L values of $\vec{k}_l$.

Error associated with $\chi_4^{SS}(t)$ could not be dealt with in an identical manner, since it is a function of the clearly correlated variables $\langle Q_S(t)\rangle$ and $\langle Q_S^2(t)\rangle$:

$$\chi_4^{SS} = N\left[\langle Q_S^2(t)\rangle - \langle Q_S(t)\rangle^2\right] , \tag{9}$$

$$\langle Q_S(t)\rangle = \frac{1}{M}\sum_{m=1}^{M}\frac{1}{N}\sum_{j=1}^{N} w_{jm}(t) ,$$

$$\langle Q_S^2(t)\rangle = \frac{1}{M}\sum_{m=1}^{M}\left(\frac{1}{N}\sum_{j=1}^{N} w_{jm}(t)\right)^2 ,$$

where $w_{jm}(t) = 1$ if $|\vec{r}_{jm}(t) - \vec{r}_{jm}(0)| < a$ and 0 otherwise, for some length a.

$\chi_4^{SS}(t)$ is itself a (scaled) sample variance of $Q_S(t)$ across M samples. The sample variance distribution is well-characterized (see, eg., `http://mathworld.wolfram.com/SampleVarianceDistribution.html`) and we can use its established (scaled) variance as the variance of $\chi_4^{SS}(t)$:

$$\sigma^2(t) = \frac{N^2(M-1)^2}{M^3}\mu_4^Q(t) - \frac{N^2(M-1)(M-3)}{M^3} \times \left(\mu_2^Q(t)\right)^2 \tag{10}$$

where $\mu_4^Q(t)$ is the fourth moment of the distribution of $Q_S(t)$ across M frames, and $\mu_2^Q(t)$ is its second moment.

To find the error associated with $\alpha(t)$, a function of the higher-order sample kurtosis of average particle displacements, we resorted to using a jackknife resampling technique[11,12]. We computed $\alpha(t)$ as:

$$\alpha(t) = \frac{3\langle\Delta r^4(t)\rangle}{5\langle\Delta r^2(t)\rangle^2} - 1 \tag{11}$$

$$\langle\Delta r^4(t)\rangle = \frac{1}{M}\sum_{m=1}^{M}\frac{1}{N}\sum_{j=1}^{N}(\vec{r}_{jm}(t) - \vec{r}_{jm}(0))^4$$

$$\langle\Delta r^2(t)\rangle = \frac{1}{M}\sum_{m=1}^{M}\frac{1}{N}\sum_{j=1}^{N}(\vec{r}_{jm}(t) - \vec{r}_{jm}(0))^2$$

We then used the jackknife variance estimate:

$$\sigma^2(t) = \frac{M-1}{M}\sum_{m=1}^{M}\left(\alpha_{(m)}(t) - \langle\alpha_{(m)}(t)\rangle\right)^2 , \tag{12}$$

where $\alpha_{(m)}(t)$ is $\alpha(t)$ computed with the m-th simulation frame removed.

E. Crystal stability tests

To test the stability of various crystal structures at any location in shape space, we replaced the particles of a well-equilibrated simulation snapshot of the relevant crystal at $\phi = 0.62$ with particles of the desired shape, while leaving particle positions and orientations unchanged. We then sampled in the isochoric ensemble to eliminate particle overlaps, isotropically enlarging the simulation box by a small scale factor every 10,000 MC sweeps if overlaps still existed. We subsequently compressed the system to some initial density between $\phi = 0.62$ and $\phi = 0.66$ in the manner detailed in Supplementary Method I A, and allowed it to slowly melt. During the melting process, system densities were decreased in increments of $\Delta\phi = 0.01$ every 10 million MC sweeps. Step size tuning was performed during the melting process to maintain sampling efficiency.

Pressure was calculated using a volume perturbation technique[13–15] that extrapolates pressure in hard particle isochoric simulations through evaluations of the volume scaling needed to cause particle-pair overlaps throughout the system. Its implementation in HOOMD-blue is detailed elsewhere[16,17]. Melting of the crystal structures was determined by eye and corroborated by melting equations of state; for each melting event, pressure exhibited behavior characteristic of a phase transition, and often showed non-monotonic behavior in the form of Mayer-Wood loops[18,19]. Three melting replicates were run for each state point.

Equations of state for each disordered, super-compressed fluid, shown in the upper left panel of Supplementary Figs. 6 and 7, were determined from system snapshots used to calculate dynamical order parameters. At each density, pressure was calculated for 100 snapshots, taken every 1 million MC sweeps. Pressures for each snapshot were then ensemble averaged, and the equation of state shows these averages and associated standard deviations of the mean at each packing fraction. Pressure is reported in reduced units: $p^* \equiv \beta p \sigma^3$, where $\beta \equiv 1/k_B T$, p is pressure, and $\sigma = 1$ is the characteristic length of our systems.

II. SUPPLEMENTARY DISCUSSION

A. Motifs in select disordered, pre-cursor, and assembled systems

Supplementary Figs. 2 and 3 show motif fraction as a function of packing fraction in two example glass-forming fluids at $(\alpha_a, \alpha_c) = (0, 0.5)$ and $(0.2, 0.5)$ respectively, as well as nearby pre-cursor fluids and assembled structures. We ran three or four replicate simulations at each density for the $(\alpha_a, \alpha_c) = (0, 0.4)$, $(0.1, 0.5)$, $(0.2, 0.4)$, $(0.25, 0.5)$, and $(0.3, 0.5)$ systems, to collect more statistics in the pre-cursor fluid regime. Motif fractions are ensemble-averaged over all fluid frames and shown with error bars indicating the associated standard deviation of the mean. For the crystal-forming fluids, motif fractions are plotted both for the pre-cursor fluid stage of those trajectories and for the crystalline stage of those trajectories, when applicable. In the case of the crystalline stage, motif fractions are ensemble-averaged over the final 5 frames of all crystallizing replicates and shown with error bars indicating the associated standard deviation of the mean. Frames in all trajectories are written at a frequency of once per 1 million MC sweeps.

Supplementary Fig. 2 is rather straight-forward, and shows that at densities relevant to crystallization, the disordered fluid at $(\alpha_a, \alpha_c) = (0, 0.5)$ contains fewer motifs associated with the diamond structure than nearby fluids that assemble into diamond, and fewer motifs associated with the dodecagonal quasicrystal than the nearby fluid that is capable of assembling into the dodecagonal quasicrystal. Thus, the disordered fluid is structurally different from nearby fluids that assemble into crystal structures, and reflects a higher competition between the face-to-face twisted (pink) motif associated with the diamond structure and the face-to-face aligned (red) motif associated with the dodecagonal quasicrystal. Note that in panel (B), at $\phi = 0.54$, the system at $(\alpha_a, \alpha_c) = (0, 0.4)$ forms a coexistence between the diamond structure and the fluid, and the crystal (dot-dash) motif fractions at this density reflect that. In both panels (B) and (C), at high enough densities, the fraction of twisted face-to-face pairwise motifs shown in dashed pink triangles is high enough to promote self-assembly into the diamond structure, at which point the aligned face-to-face motif shown in dot-dash red triangles is strongly suppressed in favor of the twisted face-to-face motif shown in dot-dash pink triangles. We observe some portion of the particles in the motif indicated by green triangles, associated with the *fcc* structure, in all systems at all densities. However, plots of $P_{obs}(f, \theta)$ for these systems show that this is essentially just an artifact of imposing cut-offs on the misorientation angle to define our motifs- these systems do not have any special spike in probability near 58°, the misorientation angle associated with the face-edge connected *fcc* motif.

Supplementary Fig. 3 is more complicated, due to the presence of more types of pairwise motifs in systems in this region of shape space, but nevertheless portrays a similar story to Supplementary Fig. 2. At densities relevant to crystallization, the disordered system at $(\alpha_a, \alpha_c) = (0.2, 0.5)$ contains fewer motifs associated with the diamond structure than the nearby fluid that assembles into diamond, fewer motifs (almost none in this case) associated with the *bcc* structure than the nearby fluid that assembles into *bcc*, and fewer motifs associated with the *fcc* structure than the nearby fluids that assemble into γ-brass and *fcc*. (Whether the system assembles into γ-brass or *fcc* seems to depend on the precise cocktail of motifs in the pre-cursor fluid at appropriate densities.) We also point out that the disordered system contains about half as many motifs associated with the dodecagonal quasicrystal than the fluid at $(\alpha_a, \alpha_c) = (0, 0.6)$, which is capable of assembling the quasicrystal. Thus, the disordered fluid is structurally different from nearby fluids that assemble into crystal structures, and reflects a higher competition between the face-to-face twisted (pink)

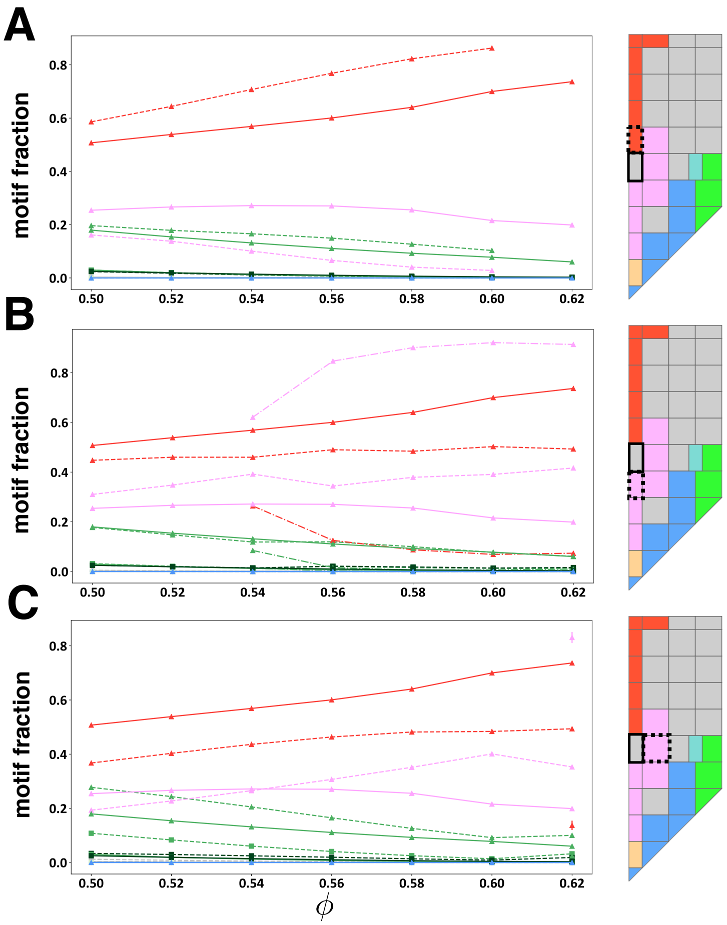

Supplementary Figure .**2:** Motif fraction as a function of packing fraction for the disordered fluid at $(\alpha_a, \alpha_c) = (0, 0.5)$ and nearby crystal-formers. Solid lines indicate motif fractions for disordered systems, dashed lines indicate motif fractions in fluids of nearby crystal-forming systems, and dot-dash lines, where shown, indicate motif fractions in assembled crystals of nearby crystal-forming systems. The location of each disordered system is outlined in a solid line in the shape space image to the right of each panel, and the location of the crystal-forming system is outlined in a dashed line. Plots show that disordered fluids and crystal-forming fluids contain different ratios of motifs in all cases, with crystal-forming fluids in general containing higher fractions of the motifs that dominate in the assembled structures. Motif fractions are shown for the disordered system at $(\alpha_a, \alpha_c) = (0, 0.5)$ and crystal-formers at (A) $(\alpha_a, \alpha_c) = (0, 0.6)$ (although this trajectory did not self-assemble into the quasicrystal during our simulation, an identical state point with different random initial conditions did; see the main text for more detail), (B) $(\alpha_a, \alpha_c) = (0, 0.4)$, and (C) $(\alpha_a, \alpha_c) = (0.1, 0.5)$.

motif associated with the diamond structure, the edge-to-face (green) motifs associated with the *fcc* structure, and the face-to-face aligned (red) motif associated with the nearby quasicrystal. In panel (C), the system at $(\alpha_a, \alpha_c) = (0.25, 0.5)$ does assemble into the γ-brass structure at $\phi = 0.6$, but the fraction of motifs in the pre-cursor fluid is quite similar to the fraction of motifs in the assembled structure, so it is difficult to delineate the crystalline motif fractions in the plot. In panel (D), we also note the non-negligible presence in the *fcc*-forming fluid of the edge-connected motifs shown as light and dark green squares, also associated with the *fcc* structure. These motifs are more suppressed in the disordered fluid at all densities.

B. Motifs across the shape landscape

Supplementary Figs. 4 and 5 show fractions of select motifs across the shape landscape in disordered or pre-cursor fluids at densities of $\phi = 0.54$ and $\phi = 0.6$, respectively. Motifs are generally more abundant

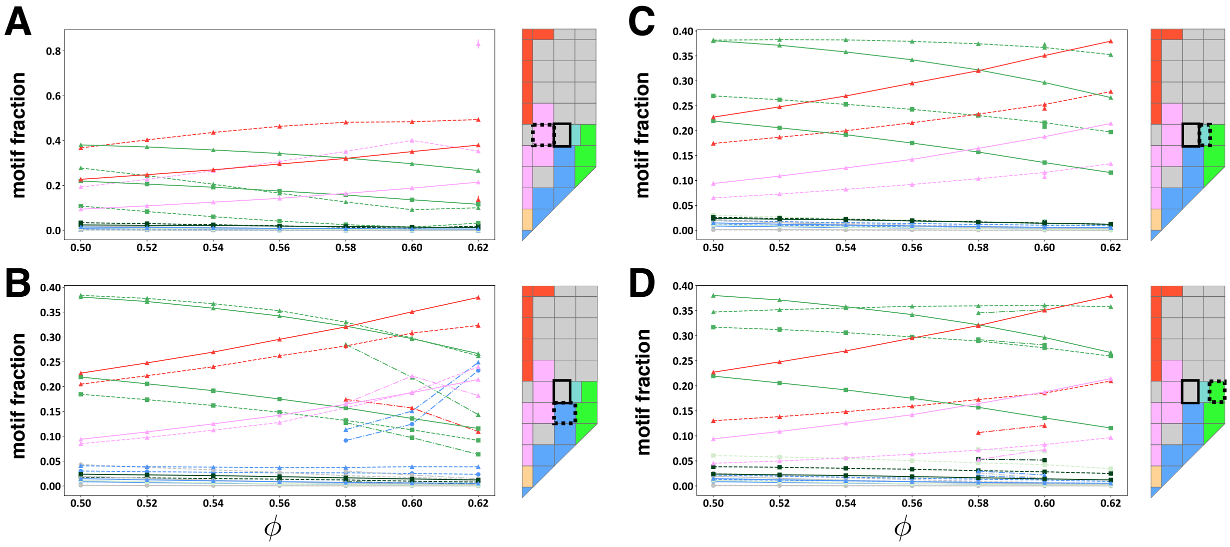

Supplementary Figure. **3:** Motif fraction as a function of packing fraction for the disordered fluid at $(\alpha_a, \alpha_c) = (0.2, 0.5)$ and nearby crystal-formers. Solid lines indicate motif fractions for disordered systems, dashed lines indicate motif fractions in fluids of nearby crystal-forming systems, and dot-dash lines, where shown, indicate motif fractions in assembled crystals of nearby crystal-forming systems. The location of each disordered system is outlined in a solid line in the shape space image to the right of each panel, and the location of the crystal-forming system is outlined in a dashed line. Plots show that disordered fluids and crystal-forming fluids contain different ratios of motifs in all cases, with crystal-forming fluids in general containing higher fractions of the motifs that dominate in the assembled structures. Motif fractions are shown for the disordered system at $(\alpha_a, \alpha_c) = (0.2, 0.5)$ and crystal-formers at (A) $(\alpha_a, \alpha_c) = (0.1, 0.5)$, (B) $(\alpha_a, \alpha_c) = (0.2, 0.4)$, (C) $(\alpha_a, \alpha_c) = (0.25, 0.5)$, and (D) $(\alpha_a, \alpha_c) = (0.3, 0.5)$.

in disordered or pre-cursor fluids in regions of shape space in which fluids tend to self-assemble into the crystals associated with those motifs. Thus, (i) the motifs associated with *bcc* are strongly suppressed in all fluids except near the $\alpha_a = \alpha_c$ line, where vertex truncation is highest, (ii) motifs associated with *fcc* are more abundant in regions with higher α_a, or edge truncation, (iii) the quasicrystal motif is more abundant near the $(\alpha_a, \alpha_c) = (0, 1)$ corner of shape space corresponding to a non-truncated tetrahedron, and (iv) the diamond motif is more abundant near the $(\alpha_a, \alpha_c) = (0, 0.35)$ location in shape space that corresponds to a vertex-truncated tetrahedron.

As density increases, the regions in which the *bcc* and *fcc* motifs are abundant become smaller and more concentrated near the $\alpha_a = \alpha_c$ line and at higher α_a values respectively. Conversely, the regions of quasicrystal and diamond structure motif abundance grow as density increases, and they grow in directions in which the corresponding crystals still self-assemble at higher density. The behavior of all regions as density increases makes sense in the context of locally dense packing arguments in hard particle fluids[26]: vertex/edge connected motifs only appear in systems in which particle vertex/edge truncation is significant, and these connection types are suppressed as density increases because face connection is enhanced as density increases. Face connection provides higher locally dense packing of particle pairs.

Note that particles on the diagonal of the shape landscape, where $\alpha_a = \alpha_c$, have octahedral symmetry rather than tetrahedral symmetry. Thus, their set of possible misorientation angles is different: in particular, it is not possible for particles with this symmetry to have misorientation angles between $\sim 65°$ and $90°$, so systems of these particles somewhat artificially display zero motifs associated with the dodecagonal quasicrystal or diamond structure.

C. Non-Gaussianity of MC sampling at short times

Here we provide some justification for the measured increase of the non-Gaussian parameter $\alpha(t)$ in our systems at short times. Monte Carlo methods can simulate a diffusive process if only local moves are made. In this case, the simulation is effectively a random walk, which becomes a Gaussian distribution as the

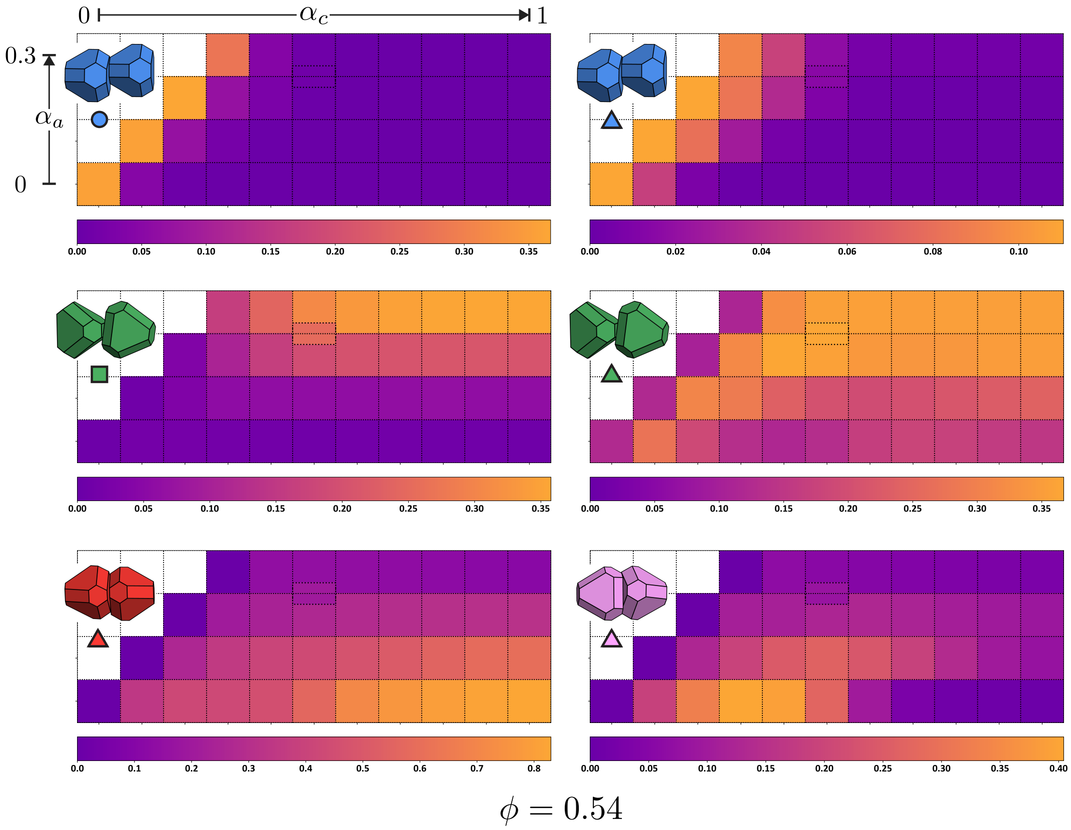

Supplementary Figure. 4: Motif fractions in disordered or pre-cursor fluids across the shape landscape at $\phi = 0.54$. Color bars below each panel show corresponding motif fraction limits. The motif whose abundance is displayed in each panel is drawn in its upper left corner, with a symbol below it indicating its connection type. α_a and α_c limits are shown for the upper left panel, and apply to all other panels. Regions of shape space are left un-filled if data at the centers of those regions is not available at $\phi = 0.54$.

number of steps taken on the walk goes to infinity. As the number of steps taken goes to zero, however, the system becomes decidedly non-Gaussian, and $\alpha > 0$.

To show this, we idealize the MC process, and compute α exactly in a toy model. First let's start in one dimension, and give our MC sampling method three options: particles can either remain in place with probability s, move to $+L$ with probability $m/2$, or move to $-L$ with probability $m/2$, with $m + s = 1$. This is technically a trinomial distribution. We will follow the treatment of a random walk in Nelson's *Biological Physics*[20]:

Let the displacement of step j be $k_j L$, where $k_j = 0$ with probability s, and $k_j = \pm 1$ with probability $m/2$. Then consider all possible trajectories of N steps:

$$\begin{aligned} \langle x_N^2 \rangle &= \langle (x_{N-1} + k_N L)^2 \rangle \\ &= \langle x_{N-1}^2 \rangle + 2L \langle x_{N-1} k_N \rangle + L^2 \langle k_N^2 \rangle \\ &= \langle x_{N-1}^2 \rangle + L^2 m \,. \end{aligned} \tag{13}$$

To get from line 2 to line 3 above, note that $\langle k_N^2 \rangle = 0s + m/2 + m/2 = m$, and that $\langle x_{N-1} k_N \rangle$ evaluates to zero. This is because x_{N-1} and k_N are uncorrelated, so we can split that average of a product into a product of averages, and $\langle k_N \rangle = 0$. As another explanation, note that for every value of x_{N-1}, there are 3 contributions to the ensemble average: $k_N = 0$ with probability s, $k_N = 1$ with probability $m/2$, and $k_N = -1$ with probability $m/2$. These average to zero.

We can compute that $\langle x_1^2 \rangle = \langle x_0^2 \rangle + L^2 m = L^2 m$, and then use the recursion relation to determine that:

$$\langle x_N^2 \rangle = NmL^2 \,. \tag{14}$$

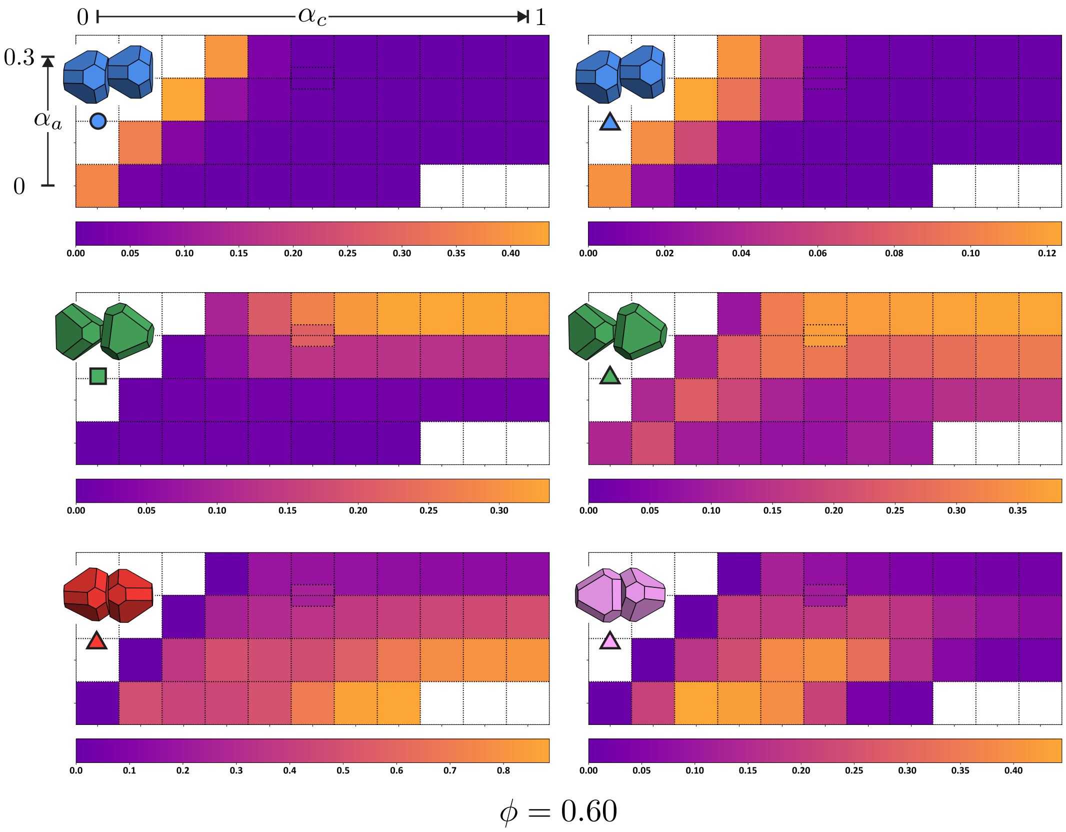

Supplementary Figure. 5: Motif fractions in disordered or pre-cursor fluids across the shape landscape at $\phi = 0.6$. Color bars below each panel show corresponding motif fraction limits. The motif whose abundance is displayed in each panel is drawn in its upper left corner, with a symbol below it indicating its connection type. α_a and α_c limits are shown for the upper left panel, and apply to all other panels. Regions of shape space are left un-filled if data at the centers of those regions is not available at $\phi = 0.6$.

We can also consider $\langle x_N^4 \rangle$:

$$\begin{aligned} \langle x_N^4 \rangle &= \langle (x_{N-1} + k_N L)^4 \rangle \\ &= \langle x_{N-1}^4 \rangle + 4L \langle x_{N-1}^3 k_N \rangle \\ &+ 6L^2 \langle x_{N-1}^2 k_N^2 \rangle + 4L^3 \langle x_{N-1} k_N^3 \rangle + L^4 \langle k_N^4 \rangle \\ &= \langle x_{N-1}^4 \rangle + L^4 m + 6L^2 m \langle x_{N-1}^2 \rangle \\ &= \langle x_{N-1}^4 \rangle + L^4 m \left(1 + 6m(N-1)\right) \ . \end{aligned} \tag{15}$$

To get from line 2 to line 3, first note that $\langle k_N^4 \rangle = 0s + m/2 + m/2 = m$. The terms that contain odd powers of k_N go to zero as per our previous argument involving $\langle x_{N-1} k_N \rangle$. Then, to evaluate $\langle x_{N-1}^2 k_N^2 \rangle$, we can again note that x_{N-1} and k_N are uncorrelated, and $\langle k_N^2 \rangle = m$, so $\langle x_{N-1}^2 k_N^2 \rangle = m \langle x_{N-1}^2 \rangle$. Line 4 proceeds from line 3 by the previous result: $\langle x_{N-1}^2 \rangle = (N-1)mL^2$.

We can compute that $\langle x_1^4 \rangle = \langle x_0^4 \rangle + L^4 m = L^4 m$, and use the recursion relation to determine that:

$$\langle x_N^4 \rangle = L^4 m \left(N + 6m \sum_{n=1}^{N} (n-1) \right) \tag{16}$$

$$\begin{aligned}
\sum_{n=1}^{N}(n-1) &= \sum_{n=0}^{N} n - \sum_{n=1}^{N} 1 \\
&= \frac{N}{2}(N+1) - N \\
&= \frac{N}{2}(N-1) \; . \qquad (17)
\end{aligned}$$

To get from line 1 to line 2, note that the first sum can be broken into pairs, $(0+N)+(1+N-1)+\ldots$. If N is odd, there are exactly $\frac{N+1}{2}$ of these pairs, for a total sum of $N\frac{N+1}{2}$. If N is even, there are exactly $\frac{N}{2}$ of these pairs, and there is a left-over term $\frac{N}{2}$ that also contributes to the sum, for a total sum of $N\frac{N}{2}+\frac{N}{2}=\frac{N}{2}(N+1)$. Thus,

$$\begin{aligned}
\left\langle x_N^4 \right\rangle &= L^4 m \left(N + 6m\frac{N}{2}(N-1) \right) \\
&= L^4 m \left(3mN^2 + N(1-3m) \right) \; . \qquad (18)
\end{aligned}$$

As $N \to \infty$, the first term dominates the expression, and $\left\langle x_N^4 \right\rangle / \left\langle x_N^2 \right\rangle^2 \to 3m^2L^4/m^2L^4 = 3$, a result that can be proven to be true for the Gaussian distribution in 1D. Let's set $N = 1$ (to mimic the short time limit in our MC simulation):

$$\begin{aligned}
\frac{\left\langle x_1^4 \right\rangle}{\left\langle x_1^2 \right\rangle^2} &= \frac{L^4 m}{L^4 m^2} \\
&= \frac{1}{m} \; . \qquad (19)
\end{aligned}$$

As $m \to 0$, or the probability of moving becomes increasingly unlikely, this expression diverges, and the distribution consequently gets increasingly "tail-heavy."

We now move to 3D by employing a few tricks:

$$\begin{aligned}
\left\langle r_1^2 \right\rangle &= \left\langle x_1^2 + y_1^2 + z_1^2 \right\rangle \\
&= 3\left\langle x_1^2 \right\rangle \\
&= 3L^2 m \; , \qquad (20)
\end{aligned}$$

and

$$\begin{aligned}
\left\langle r_1^4 \right\rangle &= \left\langle \left(x_1^2 + y_1^2 + z_1^2 \right)^2 \right\rangle \\
&= \left\langle x_1^4 + y_1^4 + z_1^4 + 2x_1^2y_1^2 + 2x_1^2z_1^2 + 2y_1^2z_1^2 \right\rangle \\
&= 3\left\langle x_1^4 \right\rangle + 6\left\langle x_1^2 \right\rangle^2 \\
&= 3L^4 m + 6L^4 m^2 \\
&= 3L^4 m(1+2m) \; , \qquad (21)
\end{aligned}$$

Thus,

$$\begin{aligned}
\frac{\left\langle r_1^4 \right\rangle}{\left\langle r_1^2 \right\rangle^2} &= \frac{3L^4 m(1+2m)}{9L^4 m^2} \\
&= \frac{1}{3m} + \frac{2}{3} \; . \qquad (22)
\end{aligned}$$

The non-Gaussian parameter α is correspondingly:

$$\begin{aligned}
\alpha &= \frac{3\left\langle r_1^4 \right\rangle}{5\left\langle r_1^2 \right\rangle^2} - 1 \\
&= \frac{1}{5m} - \frac{3}{5} \; . \qquad (23)
\end{aligned}$$

We can note several things about the above quantity. If $m = 1$, and a move is always made, $\langle r_1^4 \rangle = \langle r_1^2 \rangle^2$ and α is negative. The distribution resembles that of a Bernoulli distributed coin toss, which is known to have a negative excess kurtosis. If $m = 1/3$, $\alpha = 0$. For $m < 1/3$, however, $\alpha > 0$, and the system becomes increasingly tail-heavy. At the reasonable translation acceptance ratio $m = 0.1$, for example, $\alpha = 1.4$. Indeed, this number is comparable to values of α at small times shown in Fig. 2 in the main text.

D. Crystal stability tests

Supplementary Figs. 6 and 7 show results of stability testing of various crystal structures as functions of particle shape, at or near glass-forming locations in shape space. Near the glass-forming location at $(\alpha_a, \alpha_c) = (0, 0.5)$, shown in Supplementary Fig. 6, we studied the stability of (i) the diamond structure self-assembled at $(\alpha_a, \alpha_c) = (0, 0.4)$ as a function of increasing α_c, (ii) the dodecagonal quasicrystal self-assembled at $(\alpha_a, \alpha_c) = (0, 0.6)$ as a function of decreasing α_c, and (iii) the diamond structure self-assembled at $(\alpha_a, \alpha_c) = (0.1, 0.5)$ as a function of decreasing α_a. Near the glass-forming location at $(\alpha_a, \alpha_c) = (0.2, 0.5)$, shown in Supplementary Fig. 7, we studied the stability of (i) the diamond structure self-assembled at $(\alpha_a, \alpha_c) = (0.1, 0.5)$ as a function of increasing α_a, (ii) the *bcc* structure self-assembled at $(\alpha_a, \alpha_c) = (0.2, 0.4)$ as a function of increasing α_c, and (iii) the *fcc* structure self-assembled at $(\alpha_a, \alpha_c) = (0.3, 0.5)$ as a function of decreasing α_a.

Melting plots show the solidus line, or lowest density at which systems remain fully crystallized, and the liquidus line, or lowest density at which crystals coexist with the fluid. In all cases, the densities shown are the highest found across all replicates, since our method establishes a lower bound for melting density. Highest liquidus or solidus densities across replicates thus represent the most restrictive lower bound. Each solidus line is labeled by the crystal structure that is stable above the line. In some cases, systems undergo phase transitions during the melting process to other solids. Whether the system passes through a fluid phase during that process or undergoes a solid-solid phase transition is not shown here, because our method is not rigorous enough to determine the nature of these transitions. Instead, we simply show stability lines for all observed structures.

We now describe the phases displayed in the melting plots that are not identical to those self-assembled from the fluid. The "hR6-SbSn/hR6/oC4" melting line shows the stability of three interrelated phases, whose delineation was often not clear during the melting process itself. "hR6-SbSn" is a lower (hexagonal) symmetry diamond derivative, featuring a coordination number of 4 for all particles and squashed tetrahedral local environments. Its Pearson symbol is $hR6$, and its space group is $R\bar{3}m$, number 166. We use the short-hand hR6-SbSn because compounds of Sb and Sn have been found to crystallize into this structure[21]. "hR6" is a slightly distorted version of hR6-SbSn, with the same space group. Next nearest neighbor distances are shifted closer to each particle, such that there is no clear peak in the radial distribution function corresponding to four nearest neighbors, and instead the structure could be described with a larger coordination number of 14. "oC4" is a related structure of lower symmetry with coordination number 12. Its Pearson symbol is $oC4$, and its space group is $Cmcm$, number 63.

The "bcc (OO)" melting line corresponds to orientationally-ordered *bcc*, featuring all particles oriented in the same direction. The "bcc (OD)" melting line corresponds to orientationally-disordered *bcc*, featuring plastic-like particle orientations. Equations of state indicate a phase transition between these two phases, so we mark them distinctly. For more information regarding orientational phase transitions in hard particle systems, see Karas *et al.*[22]

The "distorted/tetragonal diamond" melting line describes systems that shear and distort, occasionally managing to form a lower symmetry tetragonal diamond derivative, when melted from cubic diamond at higher α_c. This signature appears in the pressure and indicates a phase transition between this strained or lower symmetry phase and (cubic) diamond at lower densities. Because we keep the box cubic, the phase transition is not clean, and strain occurs; however, "floppy box" simulations in these α_c regimes, in which we allow the box aspect ratio and box shear to change randomly and independently (while keeping box volume fixed), do show clear phase transitions between a lower symmetry diamond derivative phase at high packing fraction and cubic diamond at lower packing fraction. This phase transition in these systems was first observed by Cersonsky *et al.*[23] The lower-density (cubic) diamond structure melts at approximately the same density in these floppy box simulations as it does in the simulations in which we keep the box fixed and cubic.

We also used floppy box Monte Carlo to investigate the melting of the other crystals shown in these figures

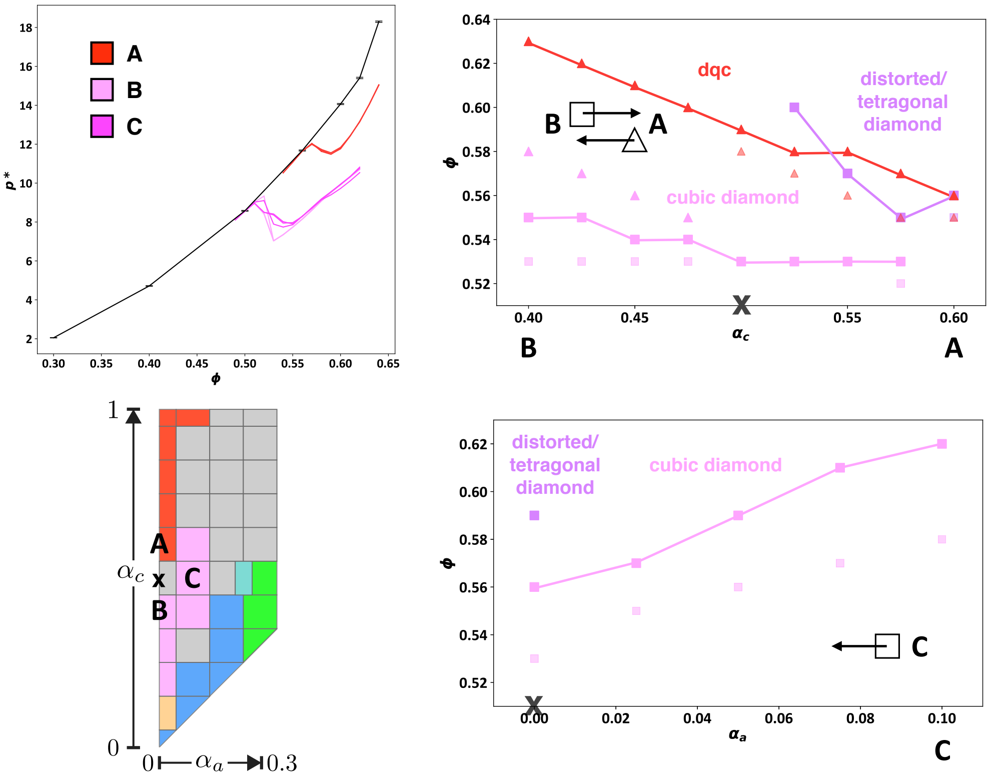

Supplementary Figure. **6:** Stability testing of crystal structures near the glass-forming state point at $(\alpha_a, \alpha_c) = (0, 0.5)$. Crystal structures tested are (A) the dodecagonal quasicrystal self-assembled at $(\alpha_a, \alpha_c) = (0, 0.6)$ as a function of decreasing α_c, (B) the diamond structure self-assembled at $(\alpha_a, \alpha_c) = (0, 0.4)$ as a function of increasing α_c, and (C) the diamond structure self-assembled at $(\alpha_a, \alpha_c) = (0.1, 0.5)$ as a function of decreasing α_a. The upper left panel shows the equation of state of the super-compressed fluid at $(\alpha_a, \alpha_c) = (0, 0.5)$ in black, and the melting equations of state of the indicated crystal structures at $(\alpha_a, \alpha_c) = (0, 0.5)$. Melting line plots show the stability of the structures initialized in each crystal as a function of α_a or α_c. Symbols at each value of α indicate the structure in which the system was initialized, as indicated by the legends embedded in the figures. Solidus lines are indicated by opaque, larger symbols, and liquidus lines are indicated by semi-transparent, smaller symbols. Symbols are colored by phase, and each phase is labeled by text above the solidus line in a matching color.

at identical locations in shape space; we did not observe any qualitatively different behavior in the melting lines or the stable structures exhibited by each system with decreasing density.

E. The identity crisis in the 423 family

Here we describe an identity crisis in a disordered system in the shape space composed of polyhedral particles in the spheric triangle invariant 423 family[24]. This set of convex polyhedra is formed by truncating the vertices and edges of an octahedron by sets of planes at varying radial distances from the polyhedron center. As we did for the 323 family described in the main text, we define truncation parameters α_a and α_c such that $(\alpha_a, \alpha_c) = (0, 0)$, denoting a cuboctahedron, $(\alpha_a, \alpha_c) = (0, 1)$, denoting a cube, $(\alpha_a, \alpha_c) = (1, 1)$, denoting a rhombic dodecahedron, and $(\alpha_a, \alpha_c) = (1, 0)$, denoting an octahedron, form the corners of this shape space. Supplementary Fig. 8A shows the full shape space, with representative polyhedra superimposed above their corresponding positions in the space. It was previously found that at certain locations in this shape space near the octahedron corner $(\alpha_a, \alpha_c) = (1, 0)$, systems failed to assemble into any ordered structure at densities ranging from $\phi = 0.50$ to 0.65, while at nearby locations, *bcc* or occasionally high-pressure Lithium formed only at high packing fractions at or above $\phi = 0.6$[1].

We investigated the region near the octahedral corner of this shape space using Monte Carlo methods

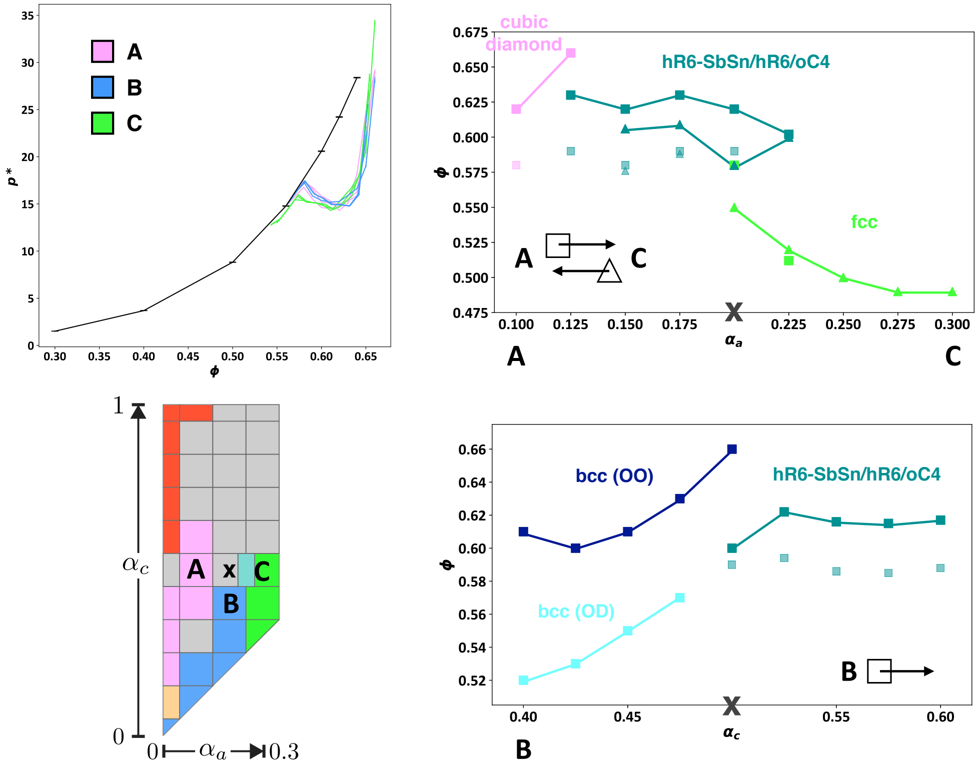

Supplementary Figure. 7: Stability testing of crystal structures near the glass-forming state point at $(\alpha_a, \alpha_c) = (0.2, 0.5)$. Crystal structures tested are (A) the diamond structure self-assembled at $(\alpha_a, \alpha_c) = (0.1, 0.5)$ as a function of increasing α_a,(B) the *bcc* structure self-assembled at $(\alpha_a, \alpha_c) = (0.2, 0.4)$ as a function of increasing α_c, and (C) the *fcc* structure self-assembled at $(\alpha_a, \alpha_c) = (0.3, 0.5)$ as a function of decreasing α_a. The upper left panel shows the equation of state of the super-compressed fluid at $(\alpha_a, \alpha_c) = (0.2, 0.5)$ in black, and the melting equations of state of the indicated crystal structures at $(\alpha_a, \alpha_c) = (0.2, 0.5)$. Melting line plots show the stability of the structures initialized in each crystal as a function of α_a or α_c. Symbols at each value of α indicate the structure in which the system was initialized, as indicated by the legends embedded in the figures. Solidus lines are indicated by opaque, larger symbols, and liquidus lines are indicated by semi-transparent, smaller symbols. Symbols are colored by phase, and each phase is labeled by text above the solidus line in a matching color.

identical to those described earlier. Our results are shown in Supplementary Fig. 8A. They agree generally with the aforementioned previous results; we found that three state points failed to assemble on the 100 million MC sweep time scale of our simulations, for densities $\phi = 0.56, 0.58, 0.6, 0.62$, and 0.64. At four state points, also simulated at $\phi = 0.56, 0.58, 0.6, 0.62$, and 0.64, only *bcc* formed at $\phi \geq 0.6$. At the remaining two state points, those that were previously found to assemble into high-pressure Lithium at $\phi = 0.61$[1], we ran five replicate simulations each at $\phi = 0.6$, 0.61, and 0.62, and indeed found that high-pressure Lithium occasionally formed at these state points only at $\phi = 0.61$. For $\phi = 0.6$ and 0.62, only *bcc* formed. At $(\alpha_a, \alpha_c) = (0.9, 0.05)$, Lithium formed in three of the five replicate simulations at $\phi = 0.61$, and transitioned to *bcc* over the course of the simulation in two of those three cases. The remaining replicate simulations assembled into *bcc* outright. At $(\alpha_a, \alpha_c) = (0.95, 0.05)$, Lithium formed in two of the five replicate simulations at $\phi = 0.61$, and transitioned to *bcc* over the course of the simulation in both cases. Two of the remaining three replicates formed *bcc* outright, while the final replicate remained disordered.

We tested for the stability of the high-pressure Lithium structure at every investigated state point by running melting simulations in which we initialized our systems in the Lithium structure at packing fractions ranging from $\phi = 0.56$ to 0.64, and subsequently sampled in the isochoric ensemble for 39 to 61 million MC sweeps or until melting into *bcc* occurred. We initialized in the Lithium structure via a compression scheme similar to that described earlier, with a few changes. We set up our particles in a high-pressure Lithium structure at very low packing fraction, then compressed quickly to the desired density with a very small translational trial move size and a much larger rotational trial move size, to ensure that the particles

remained on-lattice during the compression. We ran three replicate simulations at each state point and each density. We additionally ran one corresponding simulation at each state point and density in which we tested for the stability of *bcc* in an identical manner. We found that, on these time scales, *bcc* was stable at all densities for all state points, and Lithium was stable at a subset of (higher) densities for all state points. We found that Lithium was stable at $\phi \geq 0.58$ for $(\alpha_a, \alpha_c) = (0.85, 0.05)$, $(0.9, 0.05)$, $(0.95, 0.05)$, and $(0.95, 0.1)$; at $\phi \geq 0.6$ for $(\alpha_a, \alpha_c) = (0.85, 0.1)$, $(0.9, 0.1)$, $(0.9, 0.15)$, and $(0.95, 0.15)$; and at $\phi \geq 0.62$ for $(\alpha_a, \alpha_c) = (0.85, 0.15)$. At other densities, all replicates of Lithium melted into *bcc*. The trend is clear: as edge and vertex truncation generally increase, and thus α_a decreases and/or α_c increases, Lithium is only stable at higher and higher densities.

These assembly and melting simulations demonstrate that high-pressure Lithium can form and remain stable in this region of shape space, especially at small edge and vertex truncations, but Lithium appears generally metastable to *bcc*.

Supplementary Fig. 8B shows the results of pairwise motif identification for the disordered system at $(\alpha_a, \alpha_c) = (0.9, 0.1)$ and $\phi = 0.62$, as well as surrounding crystal structures. Motif fractions were found by averaging across all snapshots (separated by 1 million MC sweeps) for which systems were fully crystallized, or all snapshots in the case of the disordered system. The disordered system is dominated by two motifs, colored light and dark purple, that are each prevalent in nearby *bcc* (at $\phi = 0.62$) and Lithium (at $\phi = 0.61$) structures respectively. In direct parallel to the motifs that dominate the diamond structure and the dodecagonal quasicrystal within the 323 shape family studied elsewhere in this paper, we call these the "twisted" and "aligned" motifs, respectively. The "aligned" motif, detailed in row 5 in Supplementary Fig. 8B, dominates in a nearby Lithium structure and consists of two truncated octahedra face-to-face and rotated such that their truncated vertices are aligned. The "twisted" motif, detailed in row 4 in Supplementary Fig. 8B, dominates in a nearby *bcc* structure and consists of two truncated octahedra face-to-face and rotated such that they have the same orientation, and thus their bonded faces are twisted 60° with respect to each other.

Supplementary Fig. 8C shows a subset of results for alchemical Monte Carlo (Alch-MC) sampling at the disordered state point $(\alpha_a, \alpha_c) = (0.9, 0.1)$. At densities ranging from $\phi = 0.52$ to 0.64, we equilibrated our systems for 10 million MC sweeps, then allowed only the vertex truncation parameter α_a, only the edge truncation parameter α_c, or both α_a and α_c to fluctuate as thermodynamic variables for 29.7 - 40 million MC sweeps. Due to the increased computational time required for particle-particle overlap checks after every alchemical shape move attempt in these systems, we attempted shape moves with a 25% probability after every 10 MC sweeps for all vertex and vertex+edge truncation sampling simulations, rather than following the protocol described in the main text in which we sampled shape moves with a 25% probability after every MC sweep. During the edge truncation sampling simulations, we attempted shape moves with a 25% probability after every 10 MC sweeps for 26.2 - 28.5 million MC sweeps, and attempted shape moves with a 25% probability after every MC sweep for the remainder of the time. We constrained each system to only explore the area inside a square of side length $\Delta\alpha = 0.1$ centered at its initial position in shape space. Self-assembly on our simulation time scales occurred at $\phi = 0.6$ when we allowed only α_a to fluctuate, at $\phi = 0.6$ and $\phi = 0.62$ when we allowed only α_c to fluctuate, and at $\phi = 0.6$ when we allowed both α_a and α_c to fluctuate. In these simulations, disordered systems escaped into regions of larger truncation and assembled into *bcc*, with a larger fraction of their particles accordingly adopting the twisted motif. Supplementary Figs. 22, 23, and 24 respectively show the results of vertex, edge, and vertex+edge Alch-MC sampling at all densities.

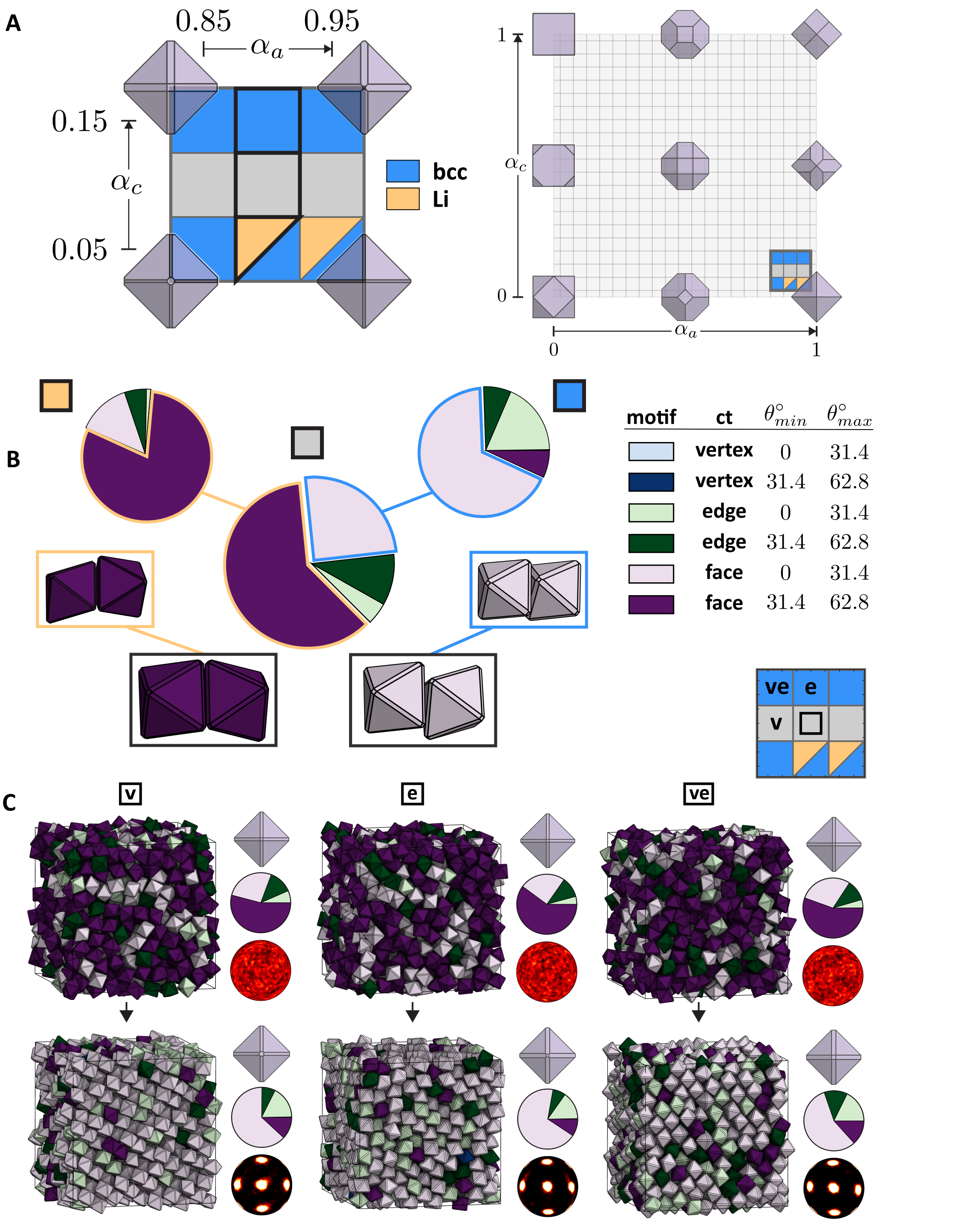

Supplementary Figure. 8: An identity crisis in the 423 shape family leads to disorder. (A) The full spheric triangle invariant 423 family, with the portion of the shape space explored in this study outlined. That portion is shown in greater detail, with sample particle shapes overlaid above corresponding regions of shape space, and regions colored according to their assembled structure. (B) Pairwise motifs in the disordered system at $(\alpha_a, \alpha_c) = (0.9, 0.1)$ compete, and are found to dominate in nearby ordered structures in shape space. Relevant locations in shape space are outlined in black in panel A. Motifs are listed in tabular form and color-coded according to their connection type **ct** and θ range. (C) The disordered system escapes its region of identity crisis and crystallizes when allowed to explore its surrounding shape space via Alch-MC. The empty square in the shape space diagram to the upper right indicates system position in shape space at the start of Alch-MC sampling, and letters indicate system position after $\sim$ 30-40 million MC sweeps of vertex truncation (**v**) sampling at $\phi = 0.6$, edge truncation (**e**) sampling at $\phi = 0.62$, or both vertex and edge truncation (**ve**) sampling at $\phi = 0.6$. System snapshots, particle shapes, pie charts of pairwise motif fractions, and bond-order diagrams are shown for initial and final frames of each Alch-MC simulation.

III. SUPPLEMENTARY FIGURES

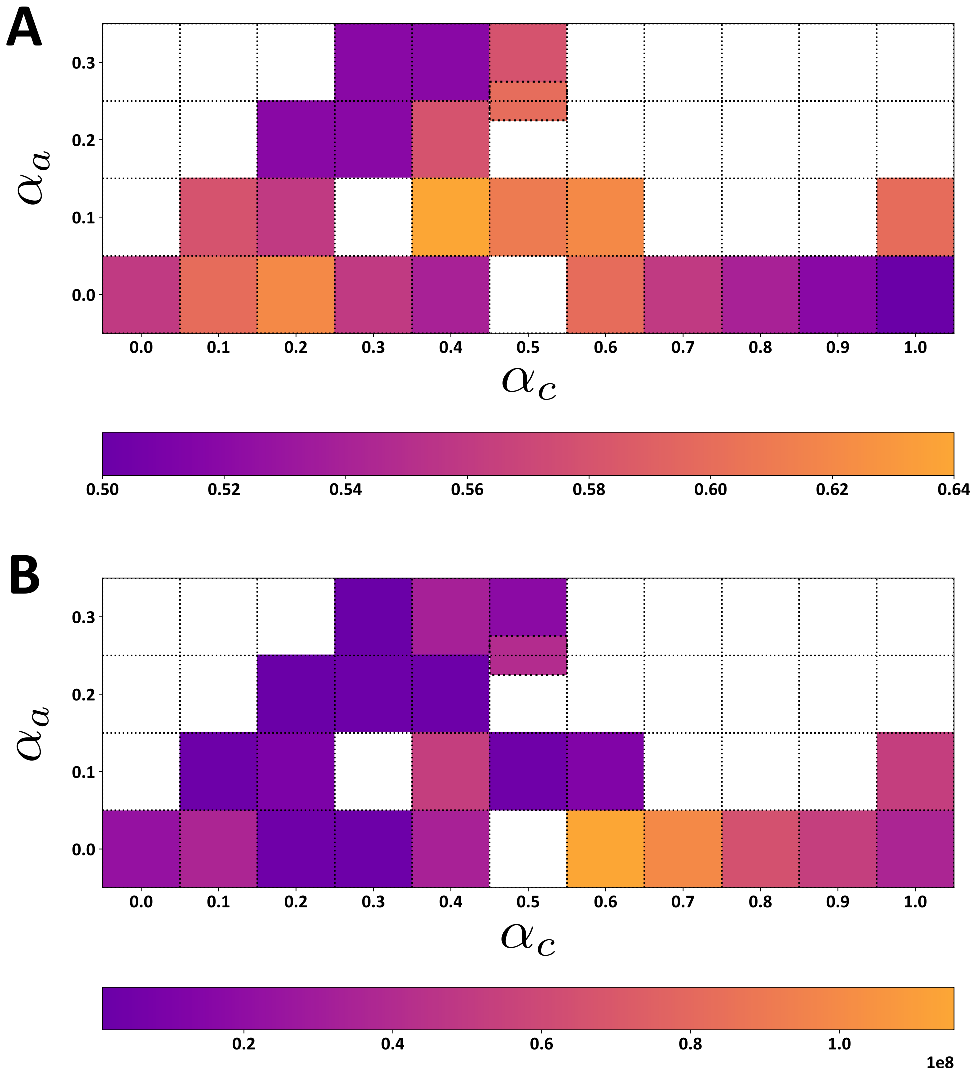

Supplementary Figure. 9: (A) Lowest packing fraction for which crystallization was observed and (B) crystallization time at that packing fraction across the shape landscape. If multiple replicates were observed to crystallize at the minimum packing fraction, the minimum crystallization time is shown. Color bars below each panel show relevant limits and scales. Squares are left uncolored if crystallization was not observed at any density at the corresponding location in shape space, or if the location in shape space is outside the bounds studied in this paper. We define crystallization time, or so-called nucleation incubation time[25], as the first frame after which approximately all crystalline particle fractions measured over the trajectory are greater than 0.1. For nucleation of the dodecagonal quasicrystal, we estimated the crystallization time by eye, corroborating our observations by calculating pressure over the trajectory (using volume perturbation methods discussed in the Supplementary Methods) when that data was available, and confirming that pressure begins to drop to its crystal value around the estimated crystallization time. For a more detailed map of the lowest packing fraction for self-assembly across this shape landscape, see Klotsa *et al.*[1]

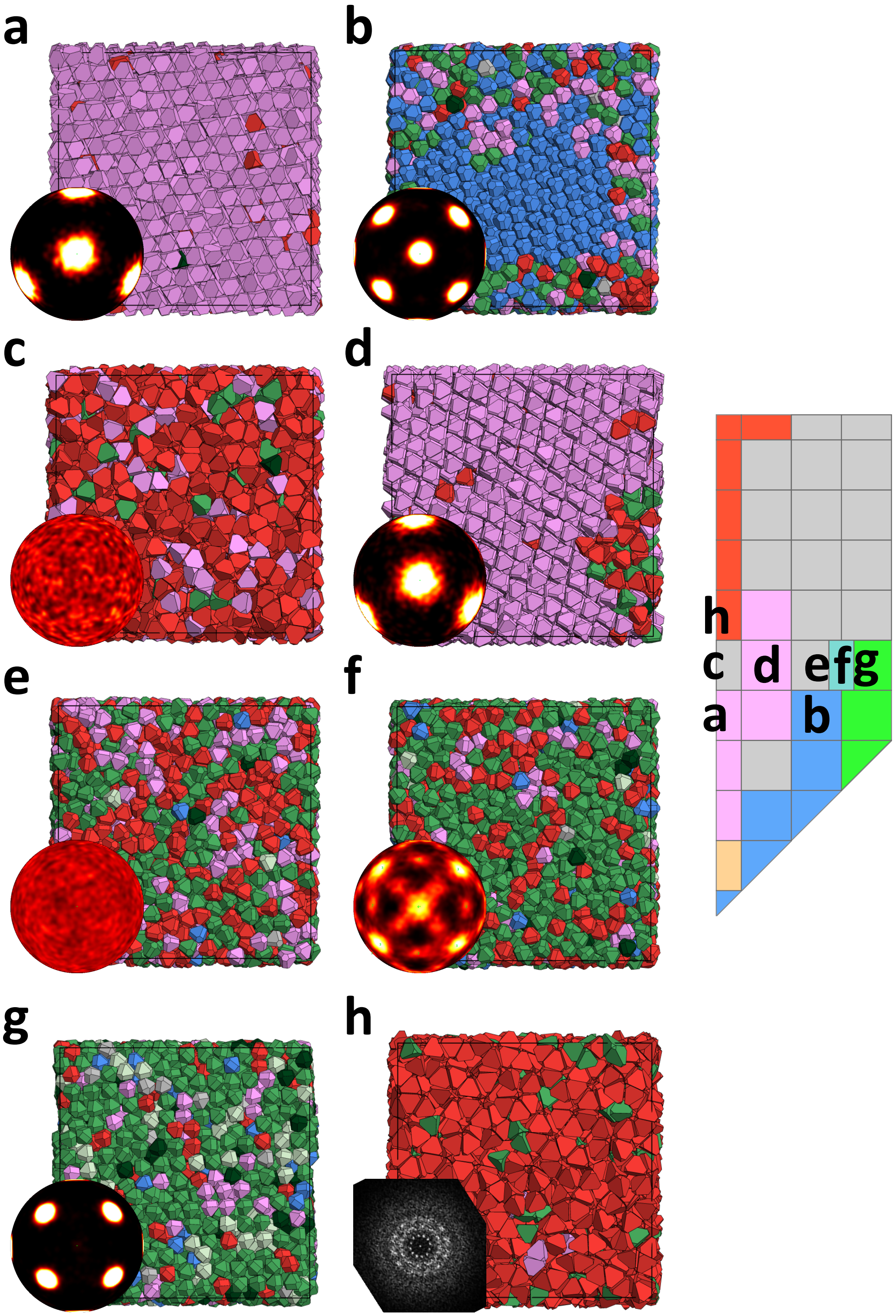

Supplementary Figure. **10:** System snapshots at packing fraction $\phi = 0.62$ of all systems considered in the main text. The exception is the γ-brass structure assembled at $(\alpha_a, \alpha_c) = (0.25, 0.5)$; it is shown for $\phi = 0.6$. Systems are located at $(\alpha_a, \alpha_c) =$ (a) $(0, 0.4)$, (b) $(0.2, 0.4)$, (c) $(0, 0.5)$, (d) $(0.1, 0.5)$, (e) $(0.2, 0.5)$, (f) $(0.25, 0.5)$, (g) $(0.3, 0.5)$, and (h) $(0, 0.6)$. Particles are colored by their associated motif. Insets show bond-order diagrams for each simulation snapshot, or a diffraction pattern in the case of the dodecagonal quasicrystal formed at $(\alpha_a, \alpha_c) = (0, 0.6)$. The snapshot shown at $(\alpha_a, \alpha_c) = (0, 0.6)$ is a dodecagonal quasicrystal self-assembled at $\phi = 0.6$ and compressed to $\phi = 0.62$, as described in the *Methods* section of the main text.

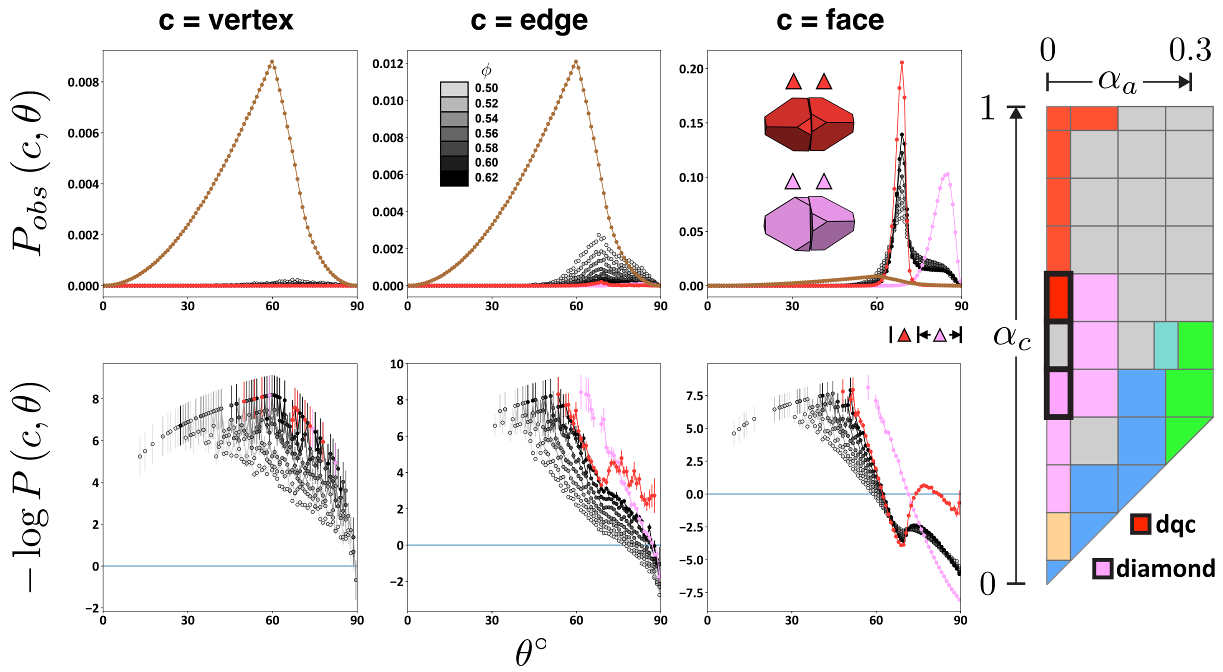

Supplementary Figure. **11:** All observed motifs in the disordered fluid at $(\alpha_a, \alpha_c) = (0, 0.5)$. Competition can be seen between face-connected aligned and twisted motifs. Motifs are prevalent in nearby diamond and dodecagonal quasicrystal (dqc) structures. Panels show probabilities of observing certain pairwise configurations, $P_{obs}(c, \theta)$, and the negative log of the normalized distributions, $-\log P(c, \theta)$, for disordered systems at the indicated densities and nearby crystals at $\phi = 0.62$. In the main text we did not show vertex- and edge-connected motifs for this system because of their improbability; we show them here.

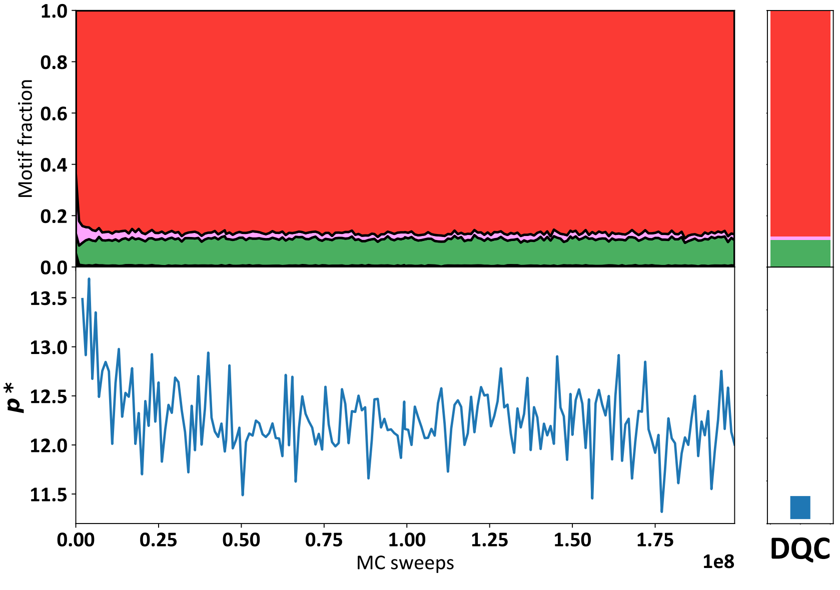

Supplementary Figure. **12:** Dimensionless pressure and motif fraction for the trajectory at $(\alpha_a, \alpha_c, \phi) = (0, 0.6, 0.6)$ in Fig. 4a in the main text, which did not assemble into the dodecagonal quasicrystal (DQC), and a crystallized DQC at this state point. Pressure and motif fraction for the DQC are shown in the right-most panel. Error bars in the pressure of the DQC are smaller than the marker size. Motif fractions of the DQC are very similar to those of the non-assembling trajectory, and pressure is lower for the assembled DQC, which is to be expected for a system that has crystallized.

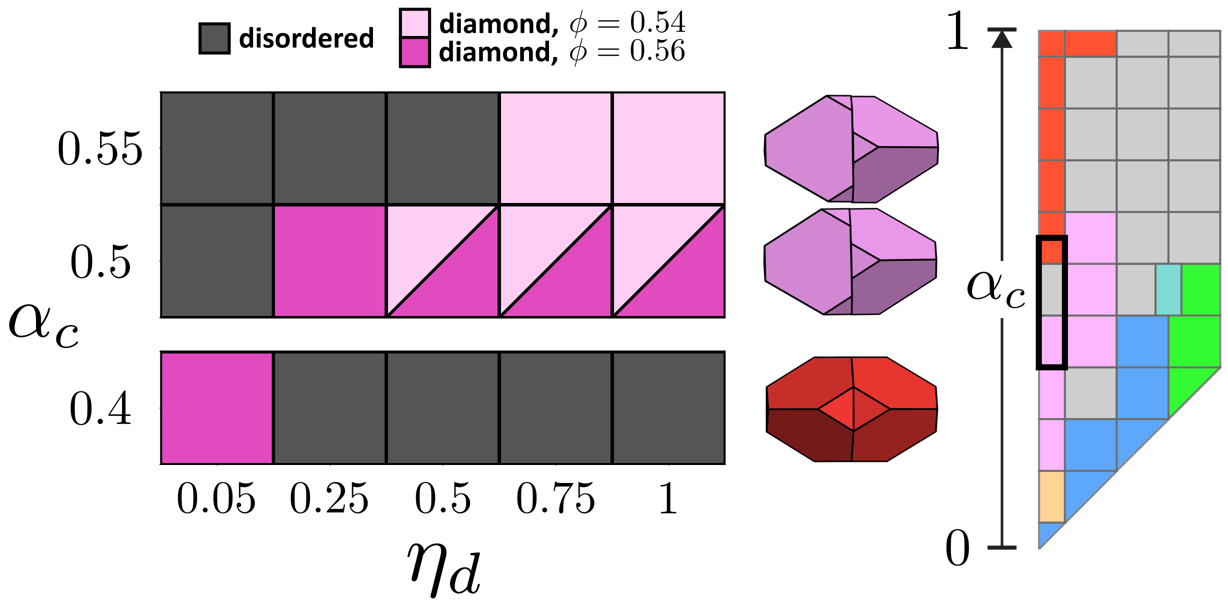

Supplementary Figure. **13:** Doping via the introduction of rigid local structural motifs influences assembly behavior, causing crystallization for systems that might otherwise vitrify, and vitrification for systems that otherwise crystallize. Systems are identified by α_c, indicating their location in shape space at $(\alpha_a, \alpha_c) = (0, \alpha_c)$, and η_d denotes the fraction of particles in the system that comprise dopant dimers. The associated dopant dimer configuration is shown to the right of each row of doping results. Boxes are colored according to whether simulations remain disordered at both $\phi = 0.54$ and $\phi = 0.56$, assemble into diamond at $\phi = 0.54$, or assemble into diamond at $\phi = 0.56$.

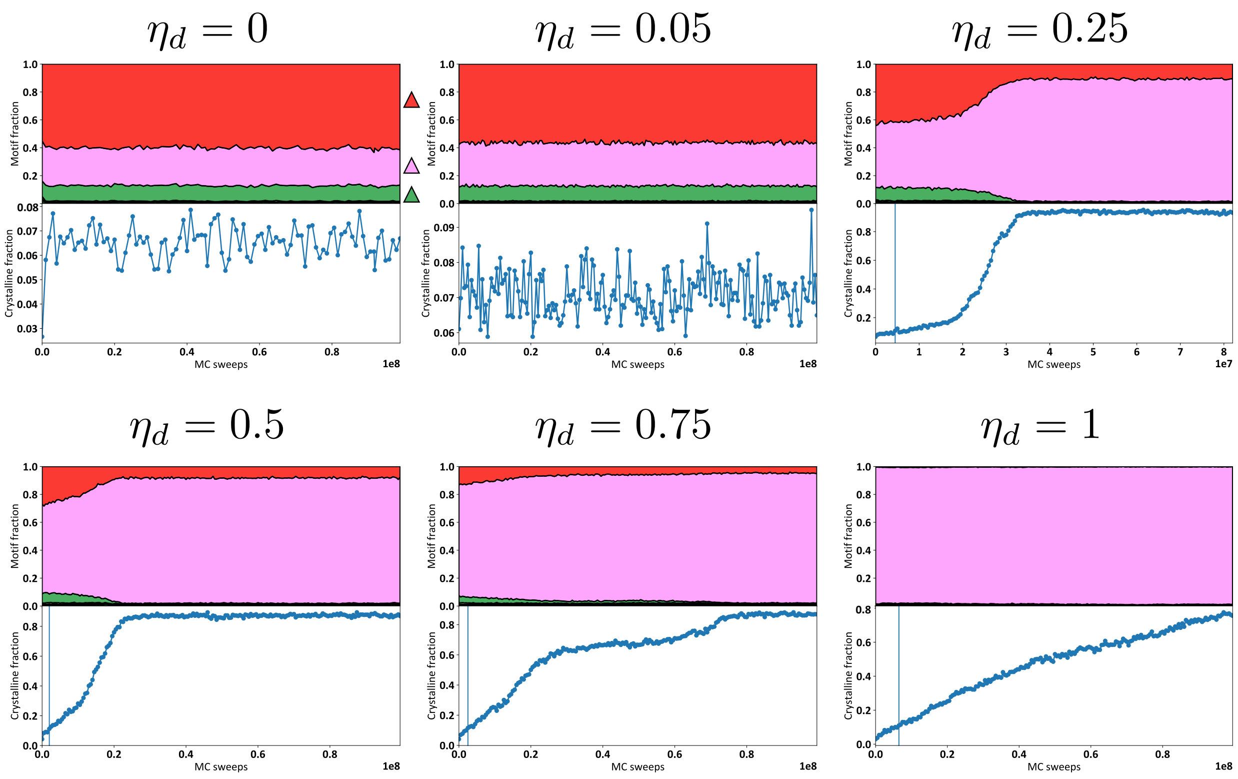

Supplementary Figure. **14:** Motif fraction and the fraction of crystalline particles in doped systems of various dimer dopant fractions η_d at $(\alpha_a, \alpha_c, \phi) = (0, 0.5, 0.56)$. The dopant dimer in this case is the face-to-face twisted motif, associated with the diamond structure. At high enough dopant dimer fraction, crystallization into diamond is promoted, and this is accompanied by a growth in the fraction of particles in the face-to-face twisted motif, shown in pink in the motif fraction plots. Vertical lines through plots of crystalline particle fraction indicate nucleation incubation time, defined in the main text. Symbols to the right of the first panel indicate the connection type of each motif shown.

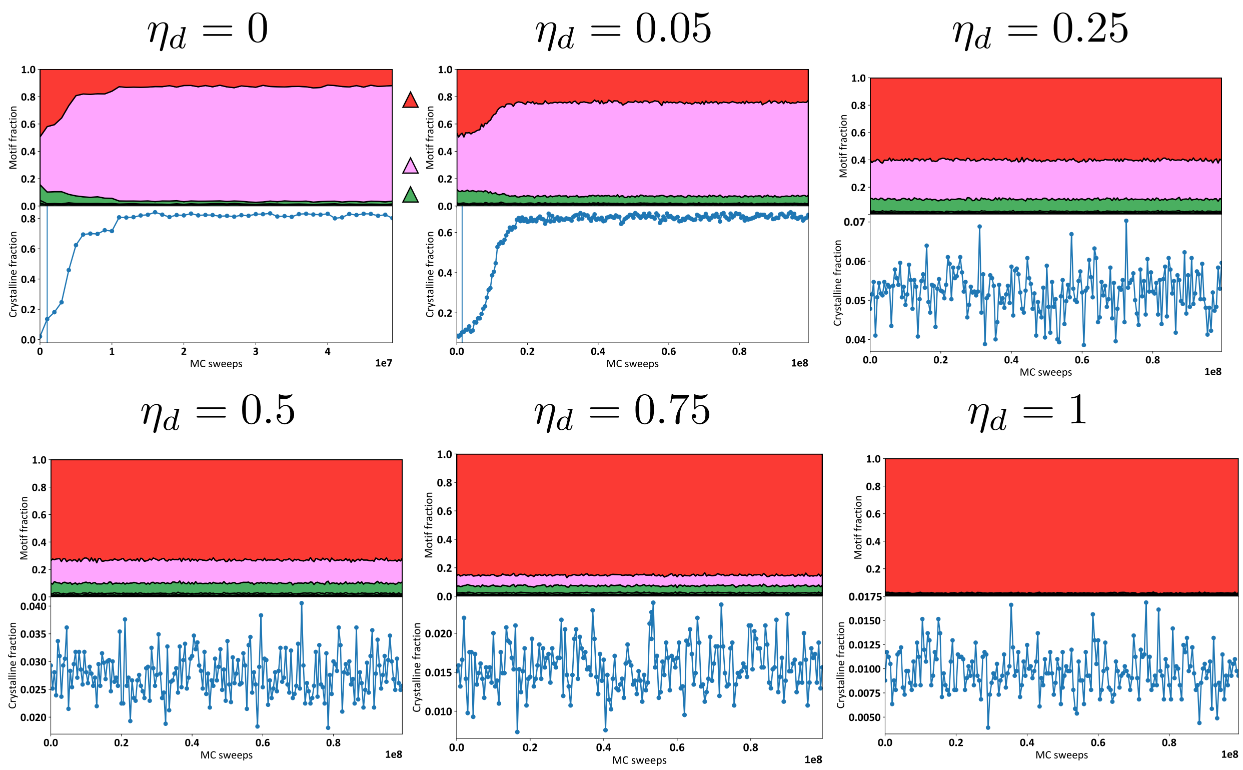

Supplementary Figure. **15:** Motif fraction and the fraction of crystalline particles in doped systems of various dimer dopant fractions η_d at $(\alpha_a, \alpha_c, \phi) = (0, 0.4, 0.56)$. The dopant dimer in this case is the face-to-face aligned motif, associated with the dodecagonal quasicrystal. At high enough dopant dimer fraction, crystallization into diamond is suppressed, and the system is dominated by the face-to-face aligned motif, shown in red in the motif fraction plots. Vertical lines through plots of crystalline particle fraction indicate nucleation incubation time, defined in the main text. Symbols to the right of the first panel indicate the connection type of each motif shown.

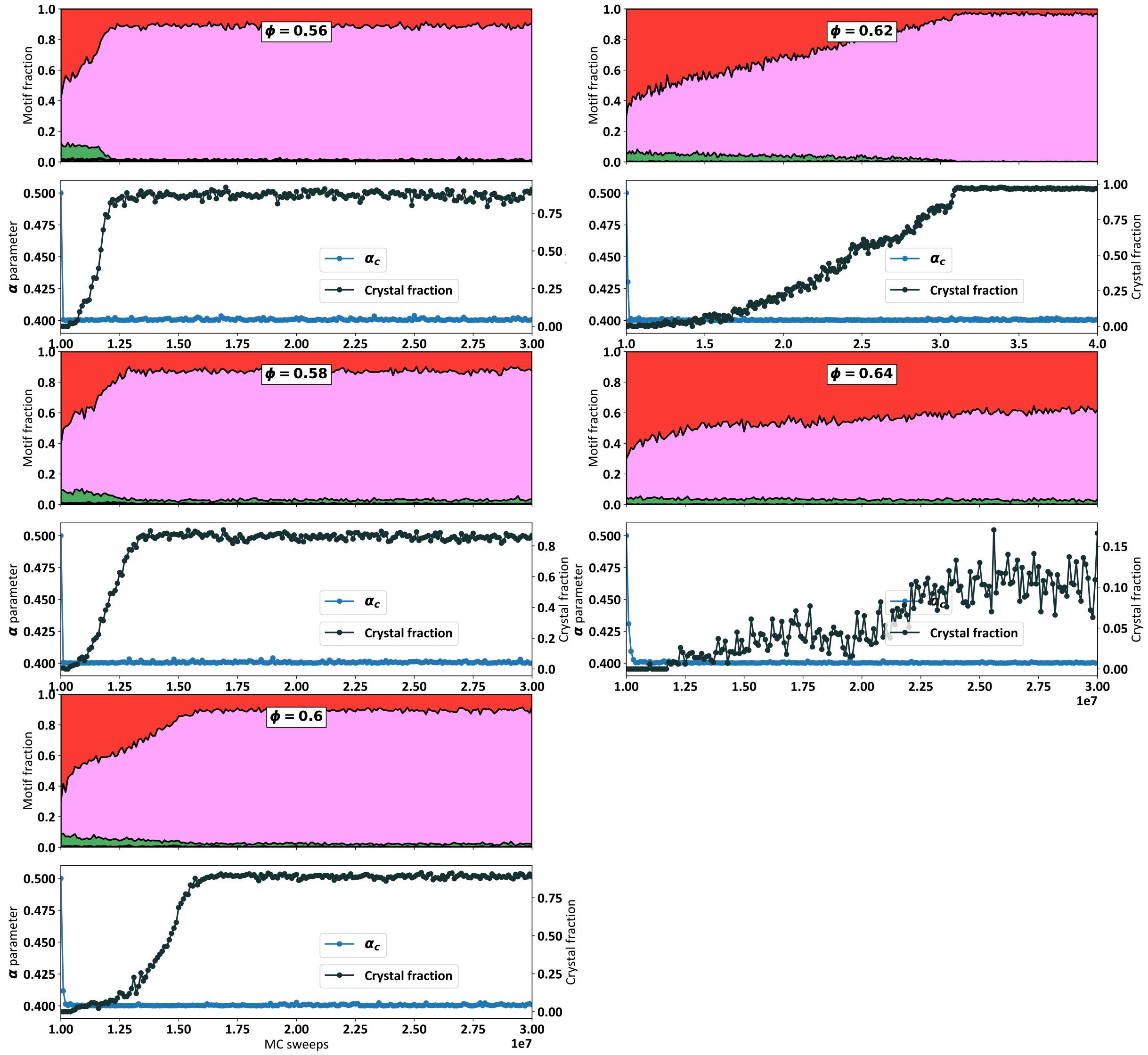

Supplementary Figure. **16:** Alchemical Monte Carlo simulations for $(\alpha_a, \alpha_c) = (0, 0.5)$ at various packing fractions ϕ, where vertex truncation is sampled as a thermodynamic variable. Plots show the fraction of particles in each motif, the truncation parameter, and the fraction of particles with a diamond crystalline environment (whose detection is detailed in the Supplementary Methods). Motif fractions are separated by black lines and colored according to the motifs identified in Fig. 3 of the main text. Regions colored gray represent (connection type, θ) regimes that were not identified with any crystal structure. Regions colored identically represent motifs characteristic of the same crystal structure that differ only by connection type. In those cases, the motif with face connection is always shown above the motif with vertex or edge connection. In most cases, systems escape the region of disorder and crystallize into the diamond structure, dominated by the pink twisted motif.

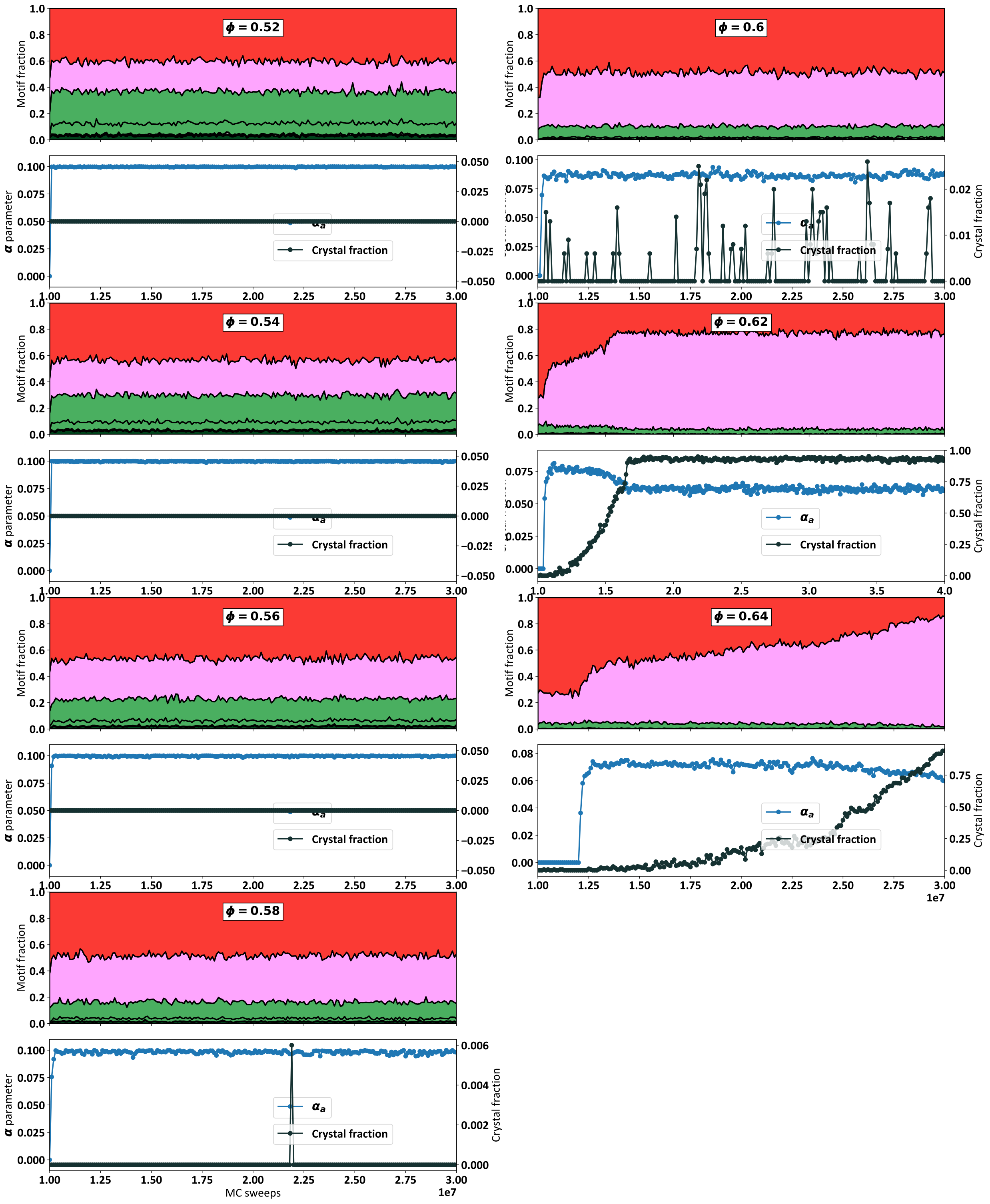

Supplementary Figure. **17:** Alchemical Monte Carlo simulations for $(\alpha_a, \alpha_c) = (0, 0.5)$ at various packing fractions ϕ, where edge truncation is sampled as a thermodynamic variable. Plots show the fraction of particles in each motif, the truncation parameter, and the fraction of particles with a diamond crystalline environment (whose detection is detailed in the Supplementary Methods). Motif fractions are separated by black lines and colored according to the motifs identified in Fig. 3 of the main text. Regions colored gray represent (connection type, θ) regimes that were not identified with any crystal structure. Regions colored identically represent motifs characteristic of the same crystal structure that differ only by connection type. In those cases, the motif with face connection is always shown above the motif with vertex or edge connection. At high enough packing fractions, systems escape the region of disorder and crystallize into a diamond-like structure dominated by the pink twisted motif. This diamond-like structure contains wurtzite-like structural motifs, as described in the main text.

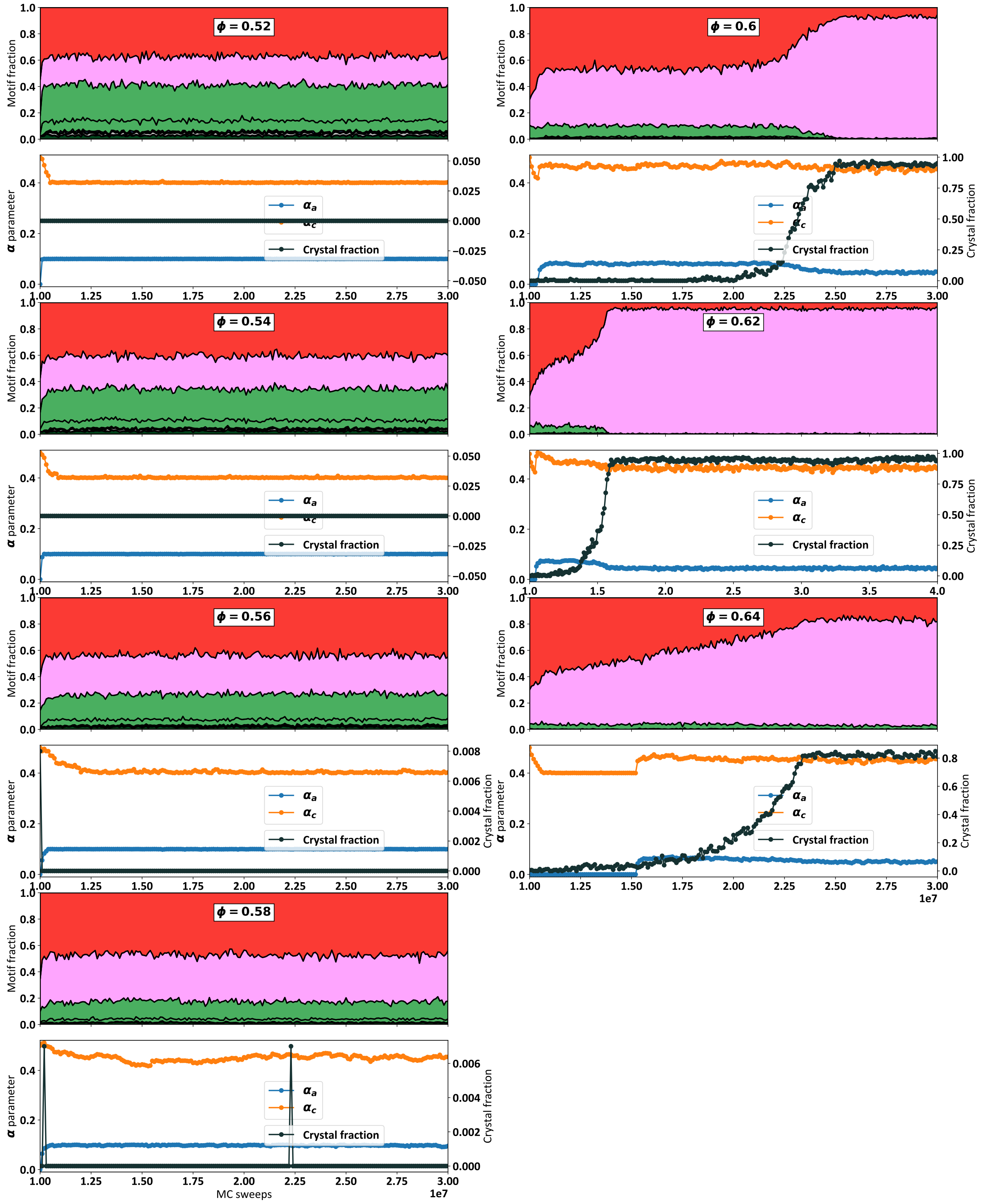

Supplementary Figure. **18:** Alchemical Monte Carlo simulations for $(\alpha_a, \alpha_c) = (0, 0.5)$ at various packing fractions ϕ, where both vertex truncation and edge truncation are sampled as thermodynamic variables. Plots show the fraction of particles in each motif, the truncation parameters, and the fraction of particles with a diamond crystalline environment (whose detection is detailed in the Supplementary Methods). Motif fractions are separated by black lines and colored according to the motifs identified in Fig. 3 of the main text. Regions colored gray represent (connection type, θ) regimes that were not identified with any crystal structure. Regions colored identically represent motifs characteristic of the same crystal structure that differ only by connection type. In those cases, the motif with face connection is always shown above the motif with vertex or edge connection. At high enough packing fractions, systems escape the region of disorder and crystallize into the diamond structure, dominated by the pink twisted motif.

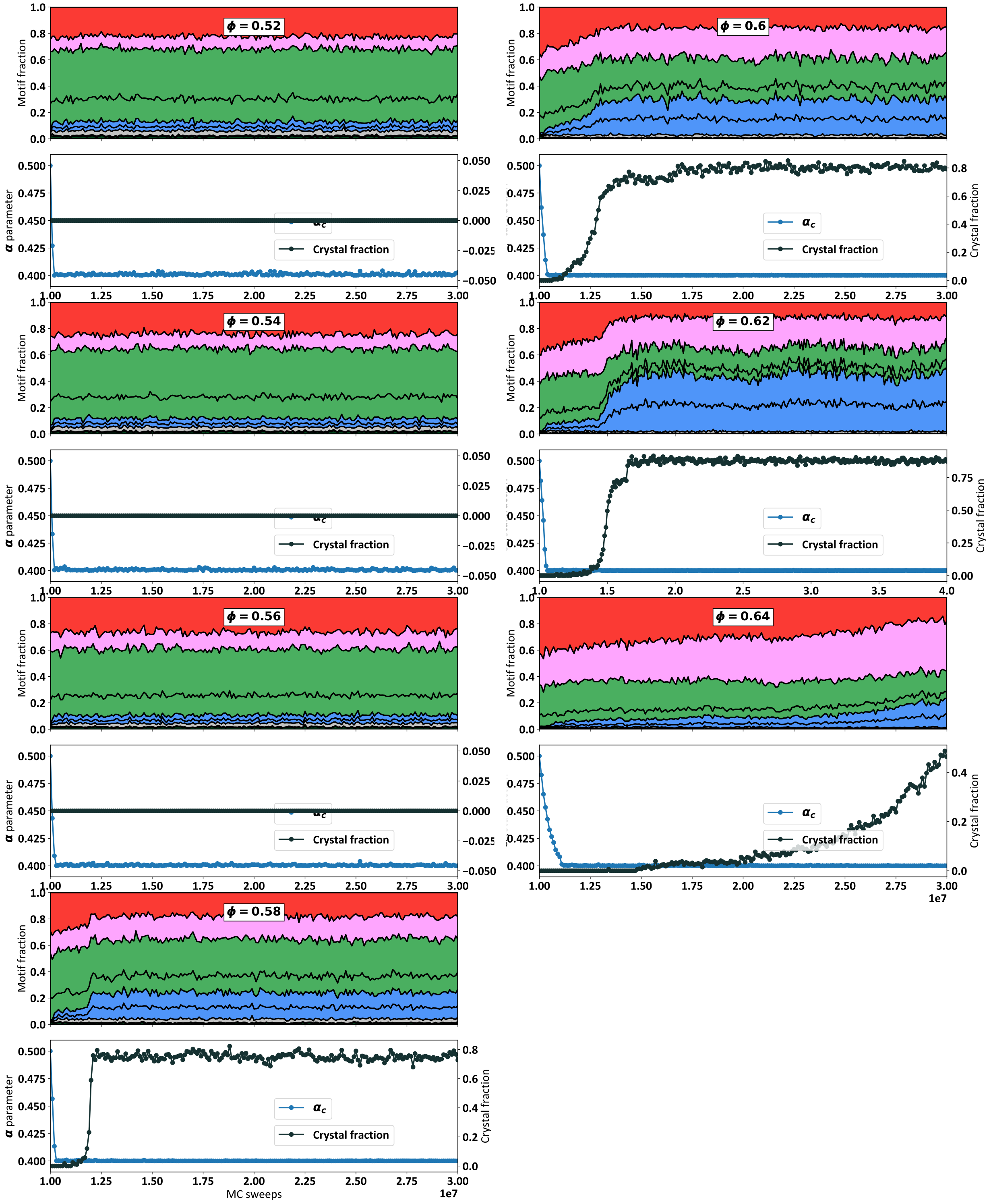

Supplementary Figure. **19:** Alchemical Monte Carlo simulations for $(\alpha_a, \alpha_c) = (0.2, 0.5)$ at various packing fractions ϕ, where vertex truncation is sampled as a thermodynamic variable. Plots show the fraction of particles in each motif, the truncation parameter, and the fraction of particles with a *bcc* crystalline environment (whose detection is detailed in the Supplementary Methods). Motif fractions are separated by black lines and colored according to the motifs identified in Fig. 3 of the main text. Regions colored gray represent (connection type, θ) regimes that were not identified with any crystal structure. Regions colored identically represent motifs characteristic of the same crystal structure that differ only by connection type. In those cases, the motif with face connection is always shown above the motif with vertex or edge connection. At high enough packing fractions, systems escape the region of disorder and crystallize into *bcc*. As packing fraction increases, this structure is increasingly dominated by the blue face-vertex connected motif shown in Fig. 3 of the main text.

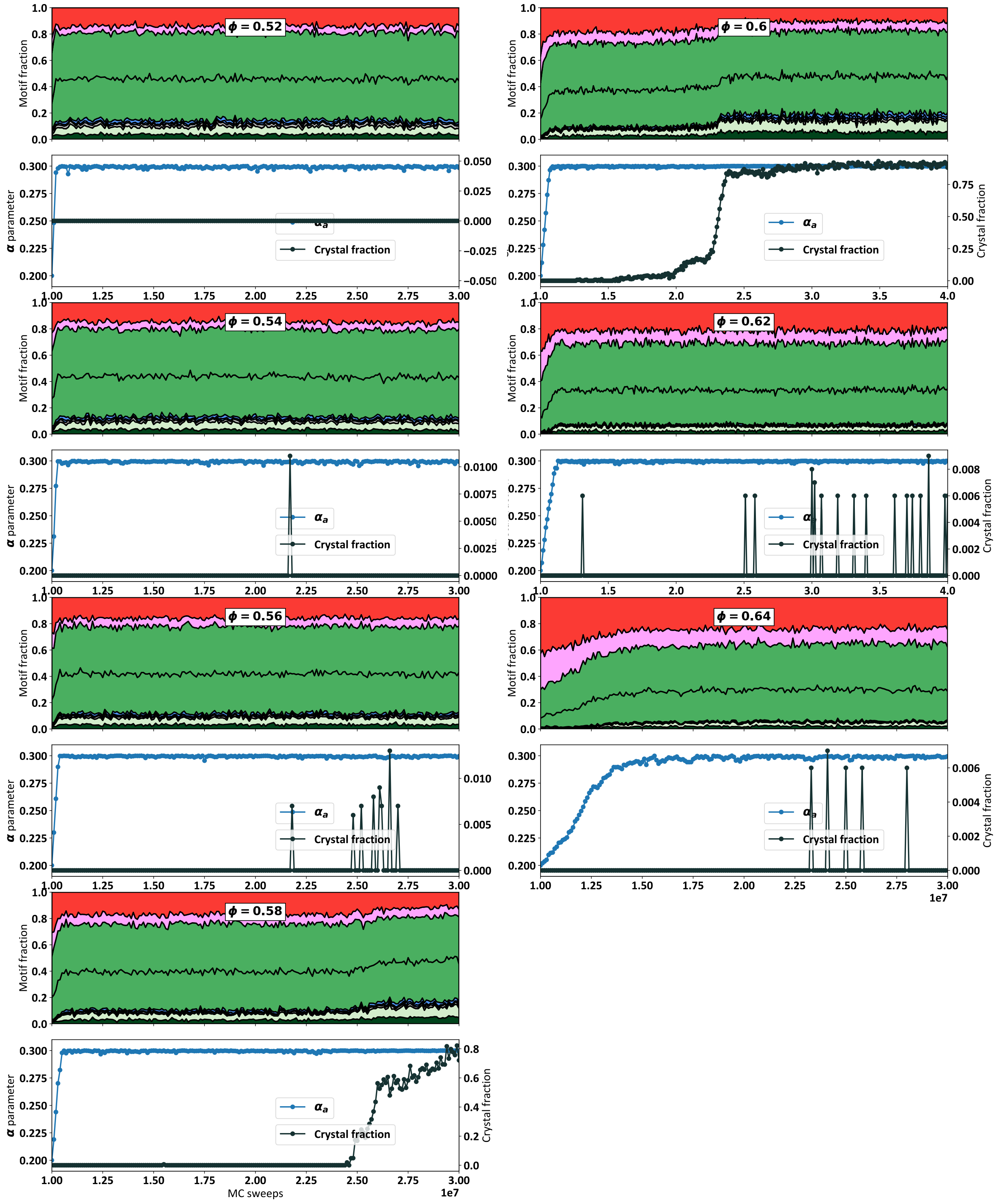

Supplementary Figure. **20:** Alchemical Monte Carlo simulations for $(\alpha_a, \alpha_c) = (0.2, 0.5)$ at various packing fractions ϕ, where edge truncation is sampled as a thermodynamic variable. Plots show the fraction of particles in each motif, the truncation parameter, and the fraction of particles with an *fcc* (or *bcc* for $\phi = 0.58$) crystalline environment. Detections of these environments are detailed in the Supplementary Methods. Motif fractions are separated by black lines and colored according to the motifs identified in Fig. 3 of the main text. Regions colored gray represent (connection type, θ) regimes that were not identified with any crystal structure. Regions colored identically represent motifs characteristic of the same crystal structure that differ only by connection type. In those cases, the motif with face connection is always shown above the motif with vertex or edge connection. At $\phi = 0.58$ and $\phi = 0.6$, systems escape the region of disorder and crystallize into *bcc* and *fcc* respectively. Interestingly, both structures in this case are dominated by the green face-edge connected motif shown in Fig. 3 of the main text. This motif is typically associated with *fcc* only.

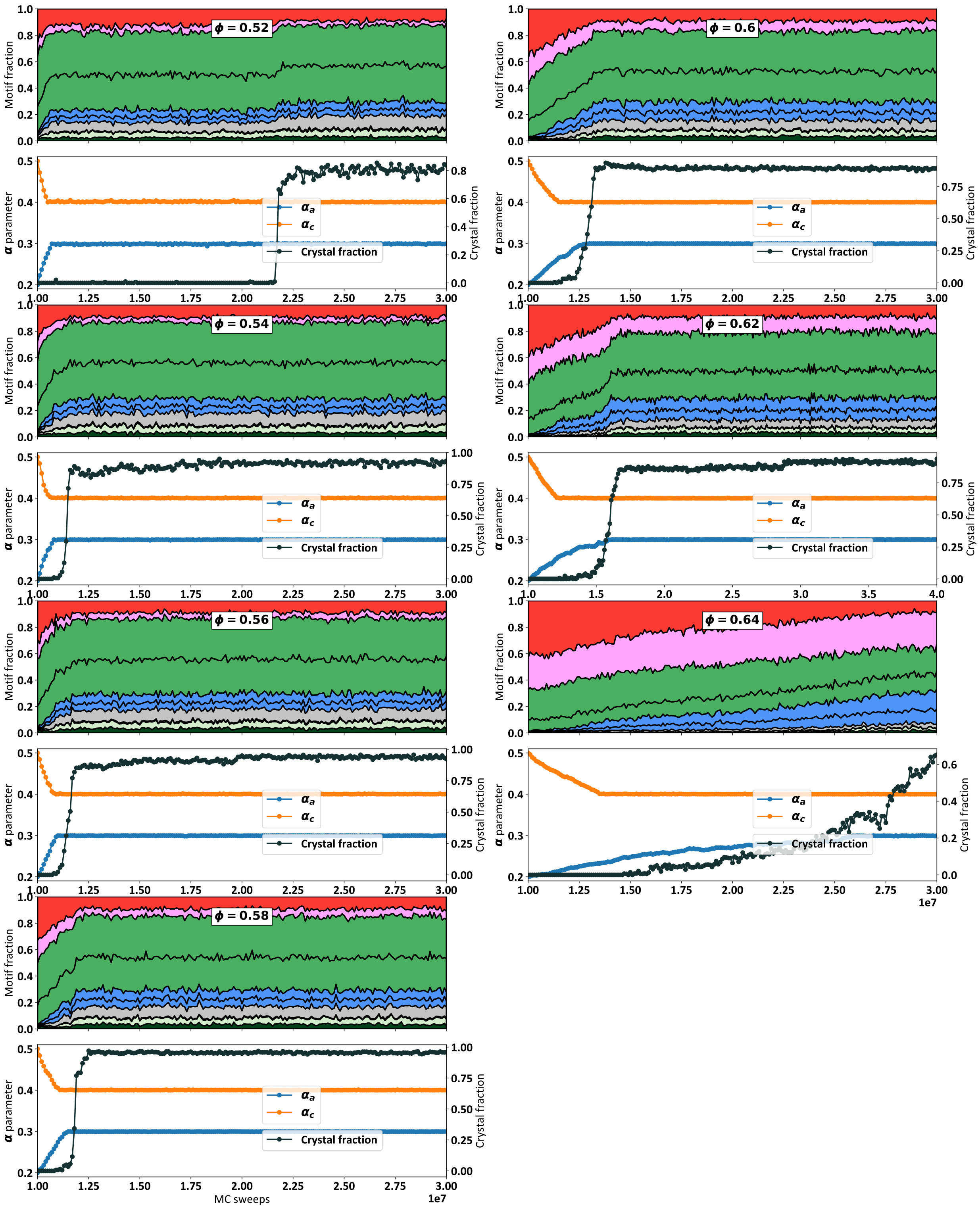

Supplementary Figure. **21:** Alchemical Monte Carlo simulations for $(\alpha_a, \alpha_c) = (0.2, 0.5)$ at various packing fractions ϕ, where both vertex truncation and edge truncation are sampled as thermodynamic variables. Plots show the fraction of particles in each motif, the truncation parameter, and the fraction of particles with an *fcc* (or *bcc* for $\phi = 0.64$) crystalline environment. Detections of these environments are detailed in the Supplementary Methods. Motif fractions are separated by black lines and colored according to the motifs identified in Fig. 3 of the main text. Regions colored gray represent (connection type, θ) regimes that were not identified with any crystal structure. Regions colored identically represent motifs characteristic of the same crystal structure that differ only by connection type. In those cases, the motif with face connection is always shown above the motif with vertex or edge connection. In all cases, systems escape the region of disorder and crystallize into *fcc* (or *bcc* for $\phi = 0.64$). *fcc* is dominated by the green face-edge connected motif shown in Fig. 3 of the main text. The *bcc* structure assembled at $\phi = 0.64$ displays an increased fraction of particles in the blue face-vertex connected motif shown in Fig. 3 of the main text, typically associated with *bcc*.

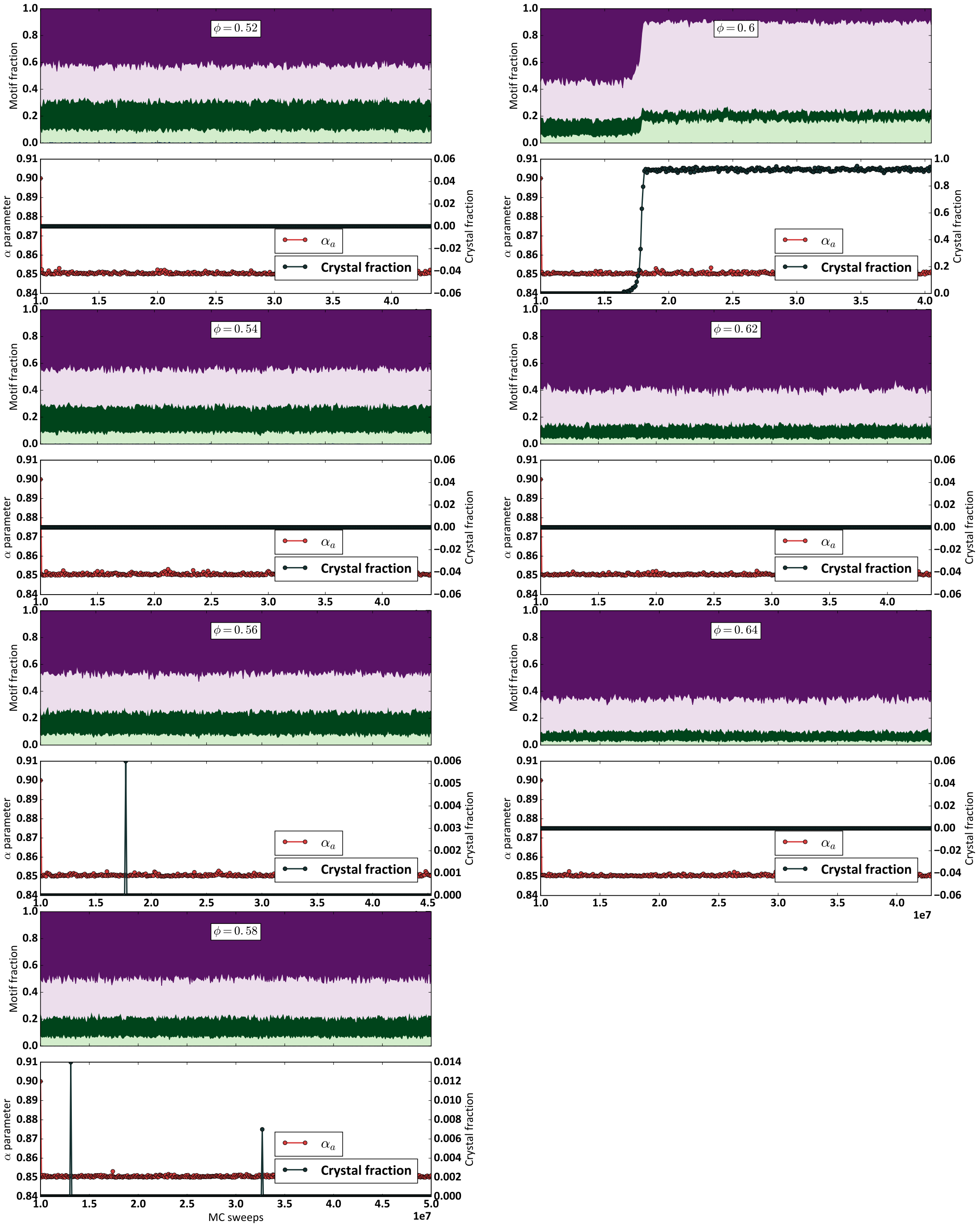

Supplementary Figure. **22:** Alchemical Monte Carlo simulations for $(\alpha_a, \alpha_c) = (0.9, 0.1)$ at various packing fractions ϕ, where vertex truncation is sampled as a thermodynamic variable. Plots show the fraction of particles in each motif, the truncation parameter, and the fraction of particles with a *bcc* crystalline environment (whose detection is detailed in the Supplementary Methods). At $\phi = 0.6$, the system increases its vertex truncation and crystallizes into *bcc*, dominated by the light purple twisted motif.

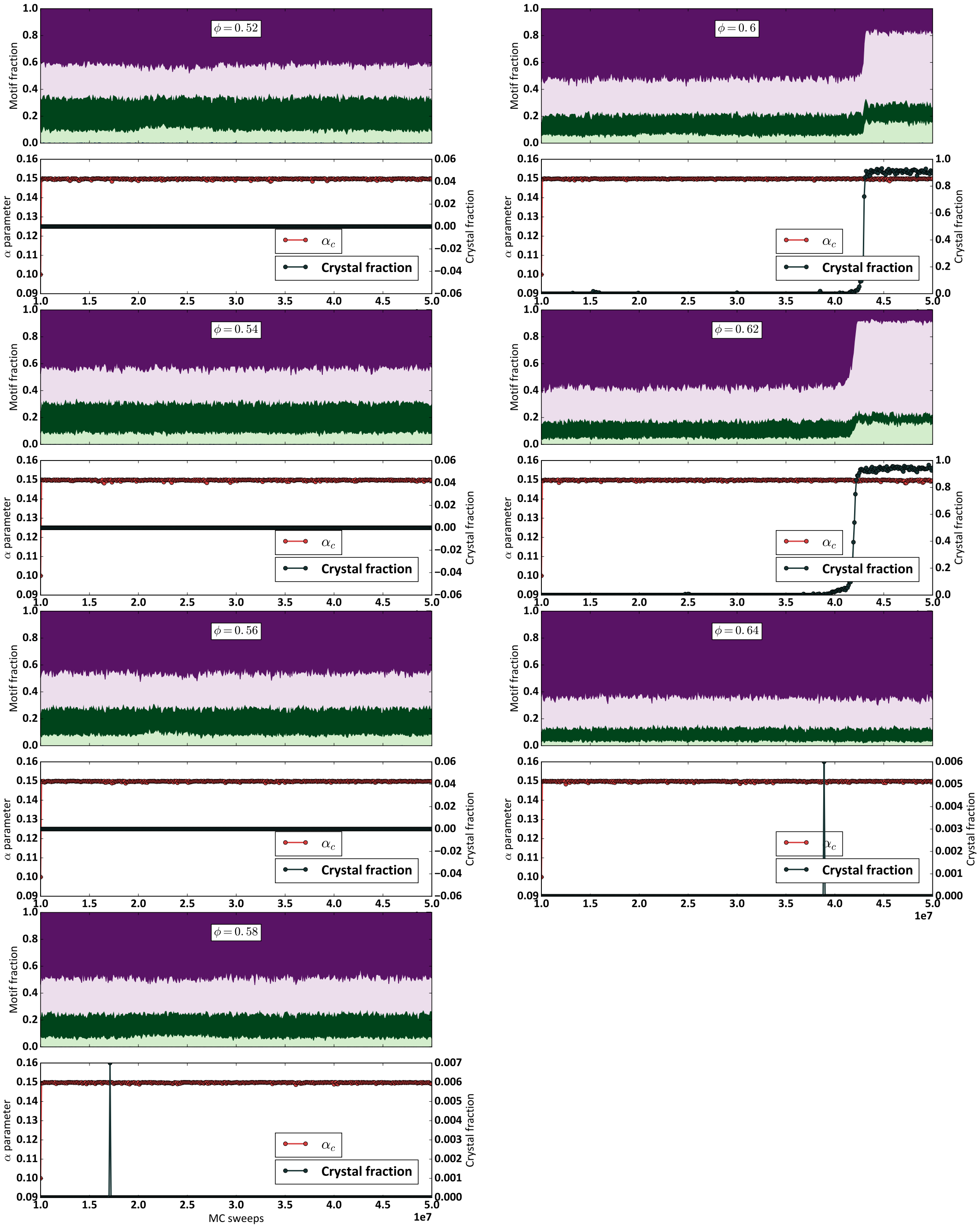

Supplementary Figure. **23:** Alchemical Monte Carlo simulations for $(\alpha_a, \alpha_c) = (0.9, 0.1)$ at various packing fractions ϕ, where edge truncation is sampled as a thermodynamic variable. Plots show the fraction of particles in each motif, the truncation parameter, and the fraction of particles with a *bcc* crystalline environment (whose detection is detailed in the Supplementary Methods). At $\phi = 0.6$ and $\phi = 0.62$, the system increases its edge truncation and crystallizes into *bcc*, dominated by the light purple twisted motif.

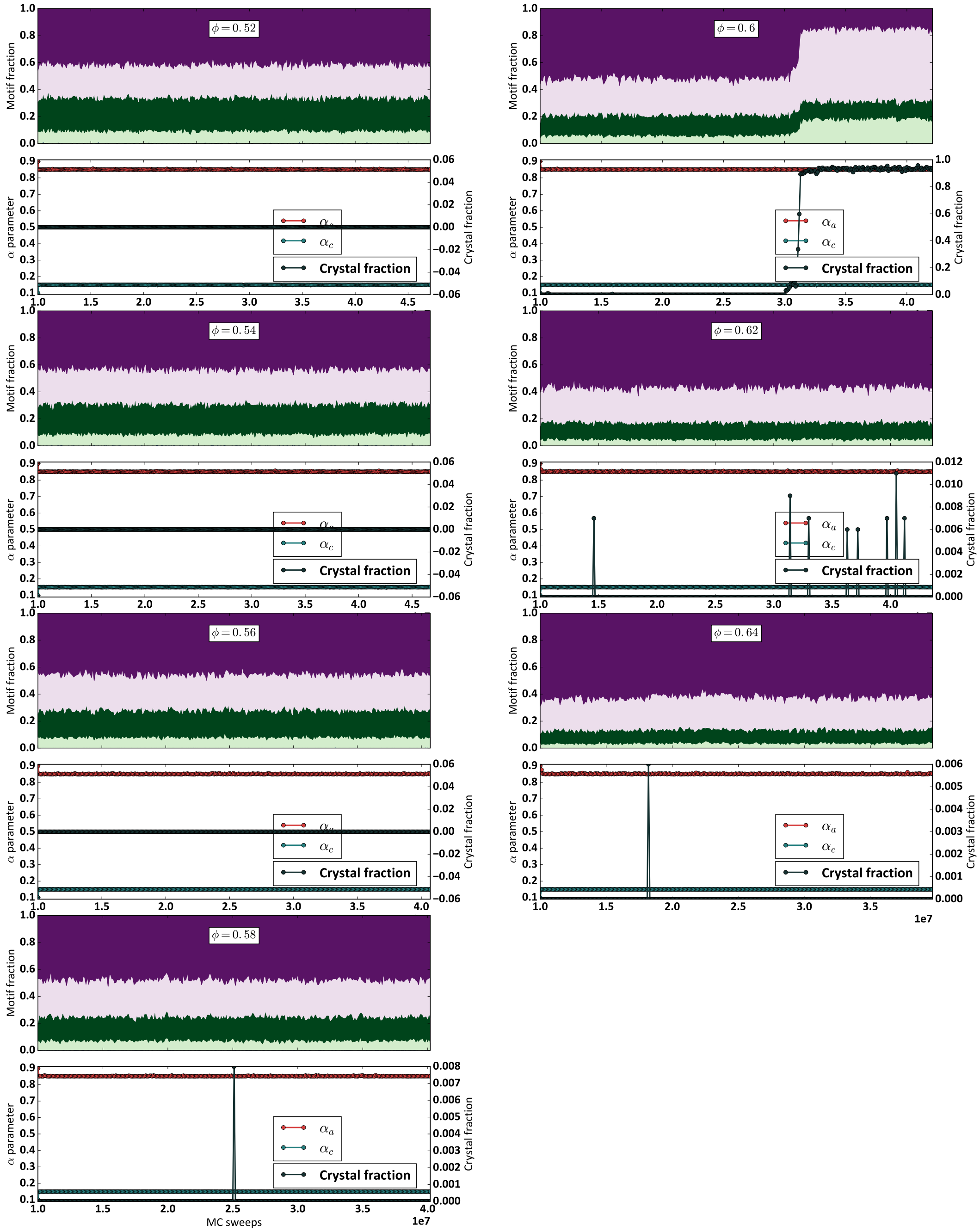

Supplementary Figure. **24:** Alchemical Monte Carlo simulations for $(\alpha_a, \alpha_c) = (0.9, 0.1)$ at various packing fractions ϕ, where both vertex truncation and edge truncation are sampled as thermodynamic variables. Plots show the fraction of particles in each motif, the truncation parameters, and the fraction of particles with a bcc crystalline environment (whose detection is detailed in the Supplementary Methods). At $\phi = 0.6$, the system increases both its vertex and edge truncation and crystallizes into bcc, dominated by the light purple twisted motif.

SUPPLEMENTARY REFERENCES

[1] Klotsa, D., Chen, E. R., Engel, M. & Glotzer, S. C. Intermediate crystalline structures of colloids in shape space. *Soft Matter* **14**, 8675–8862 (2018).
[2] Harper, E. S., Spellings, M., Anderson, J. & Glotzer, S. C. harperic/freud: Zenodo DOI release (2016).
[3] Rahman, A. Correlation in the Motion of Atoms in Liquid Argon. *Phys. Rev.* **136**, 405–411 (1964).
[4] Glotzer, S. C., Novikov, V. N. & Schrøder, T. B. Time-dependent, four-point density correlation function description of dynamical heterogeneity and decoupling in supercooled liquids. *J. Chem. Phys.* **112**, 509 (2000).
[5] Keys, A. S., Abate, A. R., Glotzer, S. C. & Durian, D. J. Measurement of growing dynamical length scales and prediction of the jamming transition in a granular material. *Nat. Phys.* **3**, 260–264 (2007).
[6] Tasios, N., Gantapara, A. P. & Dijkstra, M. Glassy dynamics of convex polyhedra. *J. Chem. Phys.* **141** (2014).
[7] Schmidt-Rohr, K. Simulation of small-angle scattering curves by numerical Fourier transformation. *J. Appl. Crystallogr.* **40**, 16–25 (2007).
[8] Hurley, M. M. & Harrowell, P. Kinetic structure of a two-dimensional liquid. *Phys. Rev. E* **52**, 1694 (1995).
[9] Kob, W., Donati, C., Plimpton, S., Poole, P. & Glotzer, S. Dynamical Heterogeneities in a Supercooled Lennard-Jones Liquid. *Phys. Rev. Lett.* **79**, 2827–2830 (1997).
[10] Marsaglia, G. Choosing a Point from the Surface of a Sphere. *Ann. Math. Stat.* **43**, 645–646 (1972).
[11] Quenouille, M. H. Approximate Tests of Correlation in Time-Series. *J. R. Stat. Soc. Ser. B* **11**, 68–84 (1949).
[12] Shao, J. & Tu, D. *The Jackknife and Bootstrap* (Springer-Verlag New York, 1995).
[13] Eppenga, R. & Frenkel, D. Monte carlo study of the isotropic and nematic phases of infinitely thin hard platelets. *Mol. Phys.* **52**, 1304–1334 (1984).
[14] Allen, M. P. Evaluation of pressure tensor in constant-volume simulations of hard and soft convex bodies. *J. Chem. Phys.* **124** (2006).
[15] Brumby, P. E., Haslam, A. J., De Miguel, E. & Jackson, G. Subtleties in the calculation of the pressure and pressure tensor of anisotropic particles from volume-perturbation methods and the apparent asymmetry of the compressive and expansive contributions. *Mol. Phys.* **109**, 169–189 (2011).
[16] Anderson, J. A., Jankowski, E., Grubb, T. L., Engel, M. & Glotzer, S. C. Massively parallel monte carlo for many-particle simulations on GPUs. *J. Comput. Phys.* **254**, 27–38 (2013).
[17] Anderson, J. A., Eric Irrgang, M. & Glotzer, S. C. Scalable Metropolis Monte Carlo for simulation of hard shapes. *Comput. Phys. Commun.* **204**, 21–30 (2015).
[18] Alder, B. J. & Wainwright, T. E. Phase Transition for a Hard Sphere System. *J. Chem. Phys.* **27**, 1208–1209 (1957).
[19] Mayer, J. E. & Wood, W. W. Interfacial tension effects in finite, periodic, two-dimensional systems. *J. Chem. Phys.* **42**, 4268–4274 (1965).
[20] Nelson, P. *Biological Physics (Updated Edition)* (W. H. Freeman, 2003).
[21] Sb (sn0.121sb0.879) crystal structure. Datasheet from "PAULING FILE Multinaries Edition – 2012", part of Springer Materials. Accessed 2018-08-01 at `https://materials.springer.com/isp/crystallographic/docs/sd_1123072`.
[22] Karas, A., Dschemuchadse, J., van Anders, G. & Glotzer, S. C. Plastic crystals of hard polyhedra and their phase behavior. In preparation.
[23] Cersonsky, R. K., Dschemuchadse, J., Antonaglia, J., van Anders, G. & Glotzer, S. C. Pressure-tunable photonic band gaps in an entropic colloidal crystal. Preprint at `http://arxiv.org/abs/1803.06388` (2018).
[24] Chen, E. R., Klotsa, D., Engel, M., Damasceno, P. F. & Glotzer, S. C. Complexity in surfaces of densest packings for families of polyhedra. *Phys. Rev. X* **4**, 011024 (2014).
[25] Russell, K. C. Grain Boundary Nucleation Kinetics. *Acta Metall.* **17**, 1123–1131 (1969).
[26] van Anders, G., Klotsa, D., Ahmed, N. K., Engel, M. & Glotzer, S. C. Understanding shape entropy through local dense packing. *Proc. Natl. Acad. Sci. U. S. A.* **111**, E4812–E4821 (2014).
